# Halogen bonding in the co-crystallization of potentially ditopic diiodotetrafluorobenzene: a powerful tool for constructing multicomponent supramolecular assemblies

**DOI:** 10.1093/nsr/nwaa170

**Published:** 2020-08-07

**Authors:** Xue-Hua Ding, Yong-Zheng Chang, Chang-Jin Ou, Jin-Yi Lin, Ling-Hai Xie, Wei Huang

**Affiliations:** Key Laboratory of Flexible Electronics (KLOFE) & Institute of Advanced Materials (IAM), Jiangsu National Synergetic Innovation Center for Advanced Materials (SICAM), Nanjing Tech University (NanjingTech), Nanjing 211816, China; Key Laboratory for Organic Electronics & Information Displays (KLOEID) and Institute of Advanced Materials (IAM), Jiangsu National Synergistic Innovation Center for Advanced Materials (SICAM), Nanjing University of Posts & Telecommunications (NUPT), Nanjing 210023, China; Key Laboratory of Flexible Electronics (KLOFE) & Institute of Advanced Materials (IAM), Jiangsu National Synergetic Innovation Center for Advanced Materials (SICAM), Nanjing Tech University (NanjingTech), Nanjing 211816, China; Key Laboratory of Flexible Electronics (KLOFE) & Institute of Advanced Materials (IAM), Jiangsu National Synergetic Innovation Center for Advanced Materials (SICAM), Nanjing Tech University (NanjingTech), Nanjing 211816, China; Key Laboratory for Organic Electronics & Information Displays (KLOEID) and Institute of Advanced Materials (IAM), Jiangsu National Synergistic Innovation Center for Advanced Materials (SICAM), Nanjing University of Posts & Telecommunications (NUPT), Nanjing 210023, China; Key Laboratory of Flexible Electronics (KLOFE) & Institute of Advanced Materials (IAM), Jiangsu National Synergetic Innovation Center for Advanced Materials (SICAM), Nanjing Tech University (NanjingTech), Nanjing 211816, China; Key Laboratory for Organic Electronics & Information Displays (KLOEID) and Institute of Advanced Materials (IAM), Jiangsu National Synergistic Innovation Center for Advanced Materials (SICAM), Nanjing University of Posts & Telecommunications (NUPT), Nanjing 210023, China; Shaanxi Institute of Flexible Electronics (SIFE), Northwestern Polytechnical University (NPU), Xi’an 710072, China

**Keywords:** halogen bond, co-crystal, physicochemical properties, supramolecular chemistry, crystal engineering

## Abstract

Halogen bonding is emerging as a significant driving force for supramolecular self-assembly and has aroused great interest during the last two decades. Among the various halogen-bonding donors, we take notice of the ability of 1,4-diiodotetrafluorobenzene (1,4-DITFB) to co-crystallize with diverse halogen-bonding acceptors in the range from neutral Lewis bases (nitrogen-containing compounds, N-oxides, chalcogenides, aromatic hydrocarbons and organometallic complexes) to anions (halide ions, thio/selenocyanate ions and tetrahedral oxyanions), leading to a great variety of supramolecular architectures such as discrete assemblies, 1D infinite chains and 2D/3D networks. Some of them act as promising functional materials (e.g. fluorescence, phosphorescence, optical waveguide, laser, non-linear optics, dielectric and magnetism) and soft materials (e.g. liquid crystal and supramolecular gel). Here we focus on the supramolecular structures of multicomponent complexes and their related physicochemical properties, highlight representative examples and show clearly the main directions that remain to be developed and improved in this area. From the point of view of crystal engineering and supramolecular chemistry, the complexes summarized here should give helpful information for further design and investigation of the elusive category of halogen-bonding supramolecular functional materials.

## INTRODUCTION

Since Lehn's famous definition of supramolecular chemistry, supramolecular synthesis is still in its formative stage as a rapidly growing field [1–3]. Important to the future of this field is the development of new synthetic methods that can produce novel supramolecular compounds with desired structures and special functionalities. Multicomponent supramolecular synthesis, for instance, the co-crystallization strategy, has a great superiority over single-component means on the regulation of both molecular arrangements and physicochemical properties [4–19].

The co-crystallization process is greatly related to molecular recognition and supramolecular self-assembly between components, which are driven by non-covalent interactions, for example, halogen bonds, hydrogen bonds, π–π stacking, van der Waals forces and so forth. Therefore, understanding of non-covalent interactions is a matter of considerable importance [20–22]. In recent years, the research focus has been extended towards halogen bonds from the well-known hydrogen bonds as they have been proven to be another powerful tool in crystal engineering and supramolecular chemistry [23–25], encompassing a wide range from fundamental studies (e.g. the nature of the halogen bond [26,27]) to materials science (photoelectric materials [28–30], liquid crystals [31,32], supramolecular gels [33], anion recognition [34,35], etc.) to biological systems [36].

**Scheme 1.**
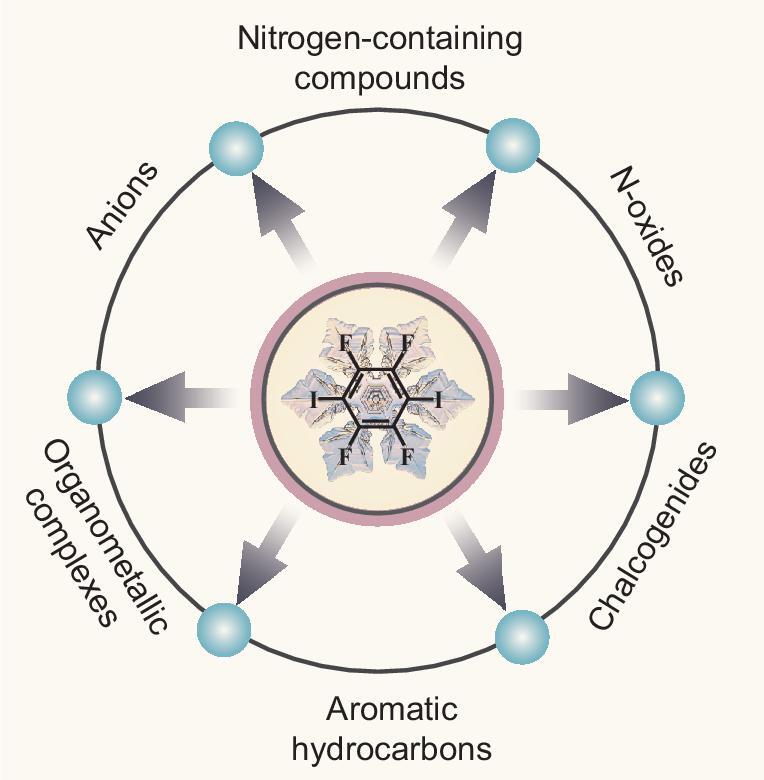
Co-crystallization of 1,4-DITFB with diverse halogen-bonding acceptors.

One common synthetic approach to achieve halogen-bonding co-crystallization systems is to utilize halogen-bonding donors and acceptors with complementary functional groups [8]. As far as halogen-bonding donors are concerned, the halogen-bonding strength depends on the electronegativity of the halogen atoms, increasing in the order of Cl < Br < I [37], and can be further enhanced by introducing electron-withdrawing groups, for example, fluorine atoms [38,39]. Consequently, perfluorinated iodobenzenes were regarded as ideal halogen-bonding donors and we have summarized the use of tritopic 1,3,5-trifluoro-2,4,6-triiodobenzene (1,3,5-TFTIB) in the design of multicomponent supramolecular complexes [40]. In this review, we will concentrate on the linear ditopic 1,4-diiodotetrafluorobenzene (1,4-DITFB), which has been more widely exploited to construct a diversity of supramolecular architectures through co-crystallizing with various halogen-bonding acceptors in the range from neutral Lewis bases (nitrogen-containing compounds, N-oxides, chalcogenides, aromatic hydrocarbons and organometallic complexes) to anions (halide ions, thio/selenocyanate ions and tetrahedral oxyanions) (Scheme 1; chemical structures of halogen-bonding acceptors seen in Supporting Information). We hope that the information given here will be useful in understanding the ‘structure–assembly–property’ correlation in this kind of halogen-bonding co-crystals as well as stimulating further research into the field of multicomponent crystalline materials.

## CO-CRYSTALLIZATION OF 1,4-DITFB WITH VARIOUS HALOGEN-BONDING ACCEPTORS

### Nitrogen-containing compounds as the halogen-bonding acceptors

#### Aromatic nitrogen-containing heterocycles

##### Pyridine derivatives.

Since pyridine is a well-known and effective halogen-bonding acceptor, various pyridine derivatives have been used as the halogen-bonding acceptors to co-crystallize with 1,4-DITFB, for instance, mono-pyridine compounds (2-methylpyridine (2MPy), 3-methylpyridine (3MPy), 4-methylpyridine (4MPy), 3,5-dimethylpyridine (DMPy), 2,4,6-trimethylpyridine (TMPy), quinolone, isoquinoline [41], phenanthridine (PhenD), benzo[*f*]quinolone (B*f*Q) and benzo[*h*]quinolone (B*h*Q) [42]). These monotopic acceptors preferably form discrete supramolecular assemblies (dimers or trimers) with 1,4-DITFB through C–I$\cdots$N halogen bonds. Therein, the pure bent 3-ring-N-heterocycles (PhenD, B*f*Q and B*h*Q) are not phosphorescent in the solid state, but their co-crystals display phosphorescence emissions with different colors (green, orange-yellow and orange, respectively), which are mainly ascribed to C–I$\cdots$π halogen bonds that make the spin–orbital coupling more efficient.

If other functional groups are included in mono-pyridine derivatives, intermolecular interactions would become complex, for example, 3-amino-pyridine (3APy), 5-amino-2-methoxypyridine (5A2MPy) [43], 4-(*N,N*-dimethylamino)pyridine (DMAPy) [44], iso-nicotinamide (INA) [45], *N*-(pyridin-3-yl)acetamide (Py3A), *N*-(pyridin-4-ylmethyl)acetamide (Py4MA), *N*-(pyridin-2-yl)acetamide (Py2A) along with pyridine-3,5-dicarboxylic acid (PyDCA) [46], methyl isonicotinate (MIN) [47], 3-(4-pyridyl)-2,4-pentanedione (PyPDONE) [48] and (*E*)-2-(((5-Methoxypyridin-2-yl)imino)methyl)phenol (MPyIMP) [49].

**Figure 1.**
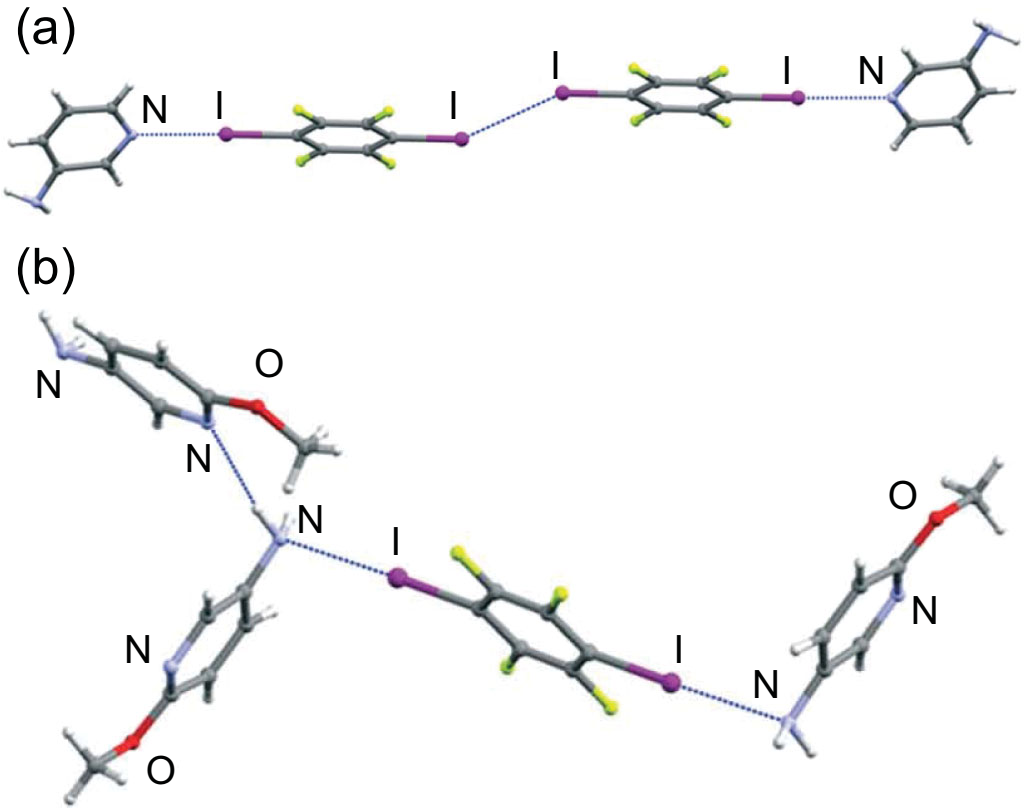
(a) Discrete tetramolecular structure in (3APy)$\cdot$(1,4-DITFB). (b) Halogen- and hydrogen-bonding interactions in (5A2MPy)_2_$\cdot$(1,4-DITFB). Reprinted with permission from reference [43]. Copyright 2016 Royal Society of Chemistry.

Among them, the reaction of aminopyridines (3APy, 5A2MPy and DMAPy) with 1,4-DITFB produces a 1 : 1 co-crystal (3APy)$\cdot$(1,4-DITFB) [43] and two 2 : 1 co-crystals (5A2MPy)_2_$\cdot$(1,4-DITFB) [43] and (DMAPy)_2_$\cdot$(1,4-DITFB) [44]. In the first case, one iodine atom from 1,4-DITFB features a strong C–I$\cdots$N halogen-bonding interaction with a pyridine nitrogen atom (d_I$\cdots$N _= 2.81 Å, ∠C–I$\cdots$N = 175°) and the other one participates in an iodine$\cdots$iodine halogen interaction (d_I$\cdots$I_ = 3.78 Å, ∠C–I$\cdots$I = 141°), giving rise to a discrete tetramer (Fig. 1a). The combination of the aforementioned halogen bonds with C–H$\cdots$F and N–H$\cdots$F hydrogen bonds, F$\cdots$F and π$\cdots$π interactions, drives the formation of a 3D supramolecular structure. In spite of sharing a similar discrete structure with 3APy, 5A2MPy shows different intermolecular interactions with 1,4-DITFB. The amino group unexpectedly turned out to be a decent halogen-bonding acceptor for the iodine atom (d_I$\cdots$N_ = 2.97 Å, ∠C–I$\cdots$N = 179°) rather than the pyridine nitrogen atom that forms the N–H$\cdots$N hydrogen bond with the NH_2_ group (Fig. 1b). The methoxy group is free and not involved in any supramolecular interactions. In (DMAPy)_2_$\cdot$(1,4-DITFB), the shortest C–I$\cdots$N halogen bond was observed between N_py_ and I atoms (d_I$\cdots$N_ = 2.67 Å, ∠C–I$\cdots$N = 179°), known in the co-crystals of fluorinated iodoarenes, to which solid-state packing effects may be a contributing factor.

**Figure 2.**
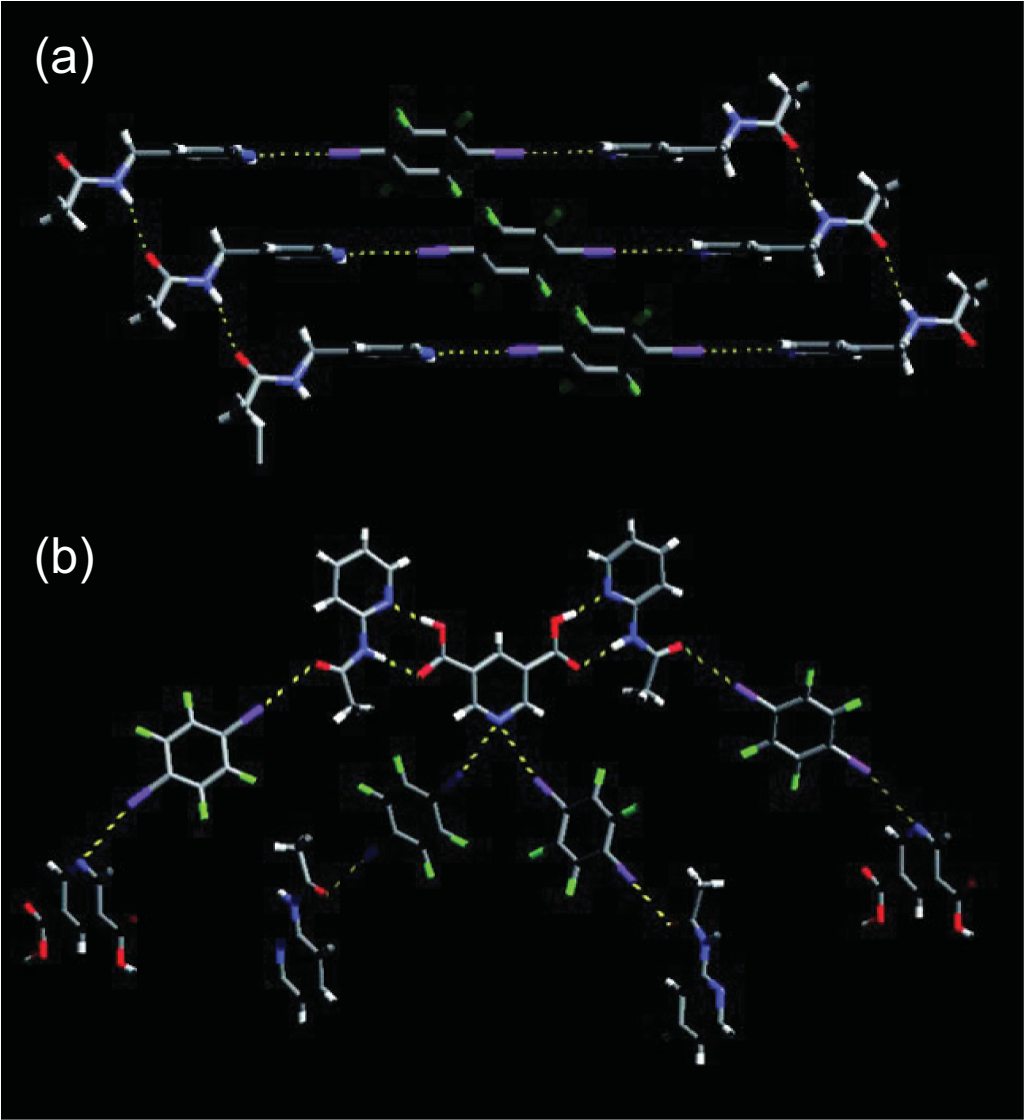
(a) Supramolecular networks in (Py4MA)_2_$\cdot$(1,4-DITFB). (b) Halogen- and hydrogen-bonding interactions in (Py2A)_2_$\cdot$(PyDCA)$\cdot$(1,4-DITFB)_2_. Reprinted with permission from reference [46]. Copyright 2008 Royal Society of Chemistry.

Several amide-substituted pyridines (INA, Py3A and Py4MA) were also utilized to react with 1,4-DITFB, resulting in three co-crystals with a 2 : 1 molar ratio [45,46]. The amide moieties are engaged in the self-complementary N–H$\cdots$O = C hydrogen-bonding interactions while pyridine N atoms are halogen bonded to I atoms, generating 3D supramolecular networks (Fig. 2a). Meanwhile, based on Py2A together with PyDCA, the first ternary co-crystal comprising 1,4-DITFB was synthesized [46], in which the carboxylic groups from PyDCA have a propensity to form a heteromeric trimer with the acetamido pyridine moiety of Py2A. It is interesting that a bifurcated halogen bond C–I$\cdots$N appears between one pyridine N atom and two 1,4-DITFB molecules, and the iodine atoms on the other side interact with the O = C moiety of the amide group (Fig. 2b).

**Figure 3.**
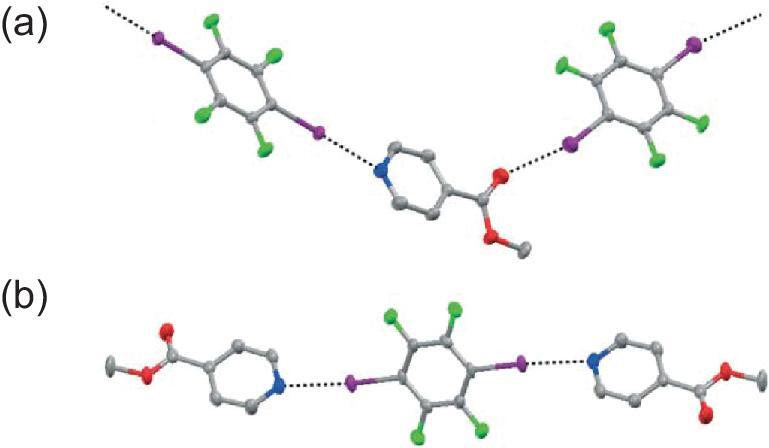
Halogen-bonding interactions in (MIN)$\cdot$(1,4-DITFB) (a) and (MIN)_2_$\cdot$(1,4-DITFB) (b). Reprinted with permission from reference [47]. Copyright 2007 Royal Society of Chemistry.

Furthermore, the behavior of MIN with an ester carbonyl was investigated in the 1 : 1 co-crystal (MIN)$\cdot$(1,4-DITFB) [47]. Both pyridine nitrogen and carbonyl oxygen atoms of MIN act as the halogen-bonding acceptors for iodine atoms, affording an infinite zigzag chain. The C–I⋯N halogen bond is stronger than C–I$\cdots$O (d_I$\cdots$N_ = 2.919 Å, ∠C–I$\cdots$N = 173.8°; d_I$\cdots$O_ = 3.045 Å, ∠C–I$\cdots$O = 165.6°), suggesting that N_py_ is the preferable binding site (Fig. 3a). This has been confirmed in the 2 : 1 co-crystal (MIN)_2_$\cdot$(1,4-DITFB), obtained by using a large excess of MIN [47]. In this case, two halogen-bonding binding sites of 1,4-DITFB are saturated by the better N atoms, leading to a discrete trimer (Fig. 3b). The similar trimeric structure was found in the co-crystallization of another carbonyl-containing PyPDONE with 1,4-DITFB [48]. Impressively, the *β*-diketone group is present as its enol tautomer and the carbonyl group is not involved in the halogen-bonding interaction, forming a six-membered ring through the very short intramolecular hydrogen bond O–H$\cdots$O with the hydroxyl group (d_H$\cdots$O_ = 1.642 Å, ∠O–H$\cdots$O = 149.08°).

**Figure 4.**
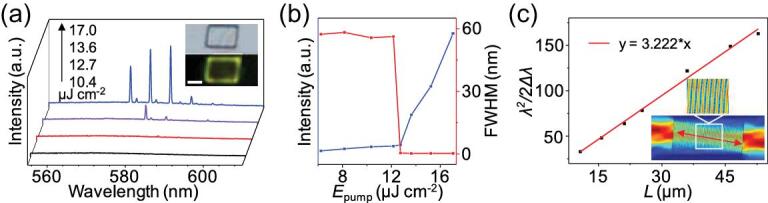
Optically pumped lasing measurements. (a) Photoluminescence (PL) spectra of a single (MPyIMP)_2_$\cdot$(1,4-DITFB) plate. Insets: Bright-field and PL images of a single plate. Scale bars are 5 μm. (b) Power-dependent profiles of the PL intensities (blue) and fwhm (red). (c) Plot and fitted curve of λ^2^/2Δλ (λ = 581 nm) vs the length of the plates. Insets: Simulated electric field intensity distributions in the plate. Reprinted with permission from reference [49]. Copyright 2018 American Chemical Society.

The incorporation of MPyIMP into the 1,4-DITFB molecule gave a 2D co-crystal microplate [49], where the pyridine N atom is halogen bonded to the iodine atom and other functional groups (−OH, −OMe, imino group) take part in the formation of hydrogen-bonding interactions. The C–I$\cdots$N halogen bonds alleviate the intermolecular charge transfer (CT), bringing about a conducive four-level energy structure for the population inversion. Optically pumped lasing measurements of the microplate reveal a self-waveguided edge emission and strong 1D field confinement as well as high-quality Fabry–Pérot (FP)-type microcavity effects so that laser oscillation is realized in the co-crystal (Fig. 4).

The self-assembly of stilbazole compounds (4-styrylpyridine (SPy) and (*E*)-4-(2-(naphthalen-1-yl)vinyl)pyridine (NVPy)) with 1,4-DITFB offered two new co-crystals with mixed stacking [50], driven by C–I$\cdots$N halogen bonds, C–H$\cdots$F hydrogen bonds and π$\cdots$π interactions. In contrast with those of individual component crystals, co-crystal (NVPy)$\cdot$(1,4-DITFB) displays new spectroscopic states and red-shifted spectra whereas (SPy)$\cdot$(1,4-DITFB) retains the energy levels of the photoluminescence states of the SPy crystal, which may be associated with the molecular configuration in crystals: NVPy is changed from twisted to planar after co-crystallization, whereas SPy retains the original planar configuration that is proposed as an ‘intramolecular emissive’ material. In particular, the aggregation-induced emission (AIE) phenomenon was discovered in (NVPy)$\cdot$(1,4-DITFB), which would enrich the multicomponent AIE family [51]. Apart from the AIE effect, light-induced fluorescence changes and mechanical responses (cracking/popping and bending/motion) can be achieved on account of the introduction of different co-formers by the co-crystal strategy [52].

Based on alkoxystilbazoles ((*E*)-4-(4-(octyloxy)styryl)pyridine (OSPy) and (*E*)-4-(4-(hexyloxy)styryl)pyridyl methacrylate (HSPyM)), two halogen-bonding trimeric complexes were reported by Resnati and Metrangolo's group [53,54], in which diiodobenzene and stilbazole molecules are packed in segregated-stacking modes. They have a monotropic liquid crystalline behavior despite the non-mesomorphic nature of the starting materials, confirming the effectiveness of halogen bonds in the construction of supramolecular mesogens.

**Figure 5.**
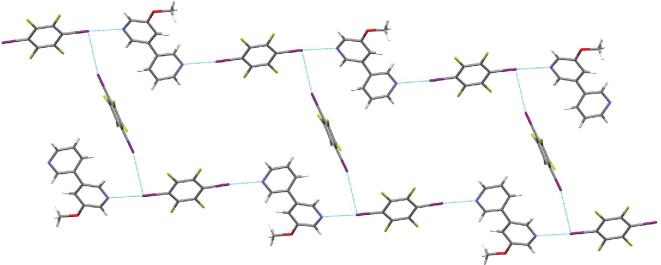
1D ladder-like halogen-bonding chains in (MeO-3,3^′^-BPy)_3_$\cdot$(1,4-DITFB)_2_ [46].

In attempts to extend this work to bipyridine compounds, a series of co-crystals were synthesized: (4,4^′^-BPy)$\cdot$(1,4-DITFB) [55,56], (2,2^′^-BPy)$\cdot$(1,4-DITFB) [57], (Na-2,2^′^-BPy)_4_$\cdot$(1,4-DITFB)_5_ and (Na-2,2^′^-BPy)_3_$\cdot$(1,4-DITFB)_2_ [58] show 1D chains of alternate bipyridine and 1,4-DITFB units while (2,4^′^-BPy)_2_$\cdot$(1,4-DITFB) [57] and (Phen-2,2^′^-BPy)_2_$\cdot$(1,4-DITFB) [58] reveal discrete termolecular structures, owing to the strong classic C–I$\cdots$N halogen bonds (4,4^′^-bipyridine (4,4^′^-BPy), 2,2^′^-bipyridine (2,2^′^-BPy), 4-(naphthalen-1-yl)-2,2^′^-bipyridine (Na-2,2^′^-BPy), 2,4^′^-bipyridine (2,4^′^-BPy) and 4-(phenanthren-9-yl)-2,2^′^-bipyridine (Phen-2,2^′^-BPy)). Moreover, based on 4-phenyl-2,2^′^-bipyridine (Ph-2,2^′^-BPy), two polymorphic forms of (Ph-2,2^′^-BPy)$\cdot$(1,4-DITFB) were obtained under different crystallization conditions (form A in orthorhombic space group *Pna*2_1_ and form B in monoclinic *P*2_1_/*c*) [58]. Form A possesses an infinite halogen-bonding chain but form B is provided with a tetrameric structure. In contrast, the structure of (MeO-3,3^′^-BPy)_3_$\cdot$(1,4-DITFB)_2_ (3-methoxy-3,3^′^-bipyridine (MeO-3,3^′^-BPy)) is complicated by the presence of halogen$\cdots$halogen bonds, wherein C–I$\cdots$N halogen-bonding chains are linked together through I$\cdots$I interactions offering ladder-like supramolecular motifs (Fig. 5) [46]. Although C–I$\cdots$N halogen bonds are the main driving forces in these co-crystals above, crystal-structure analyses make clear the significance of C–H$\cdots$F hydrogen bonds and/or π$\cdots$π interactions in directing the 3D packing.

**Figure 6.**
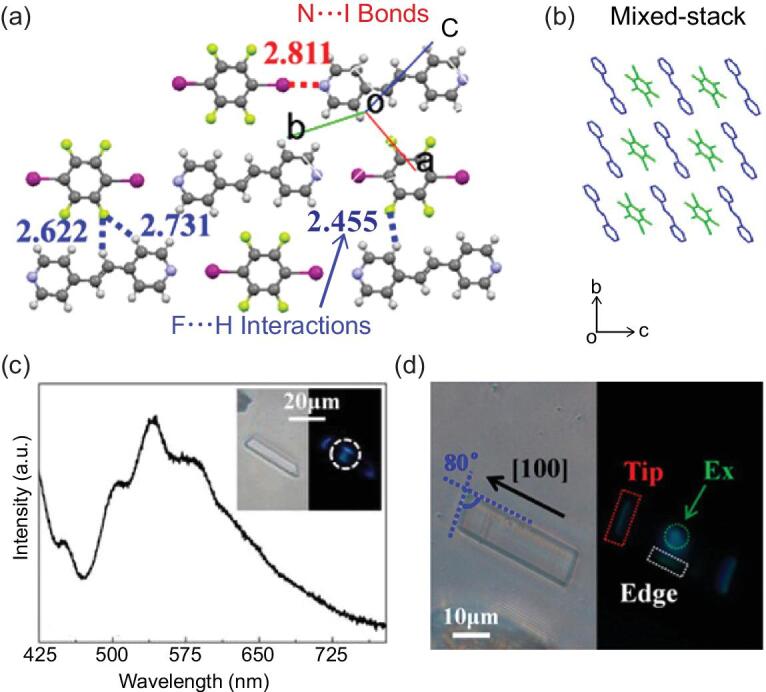
(a) Intermolecular interactions and molecular packing structures of (B4PyEe)$\cdot$(1,4-DITFB). (b) Schematic diagrams of mixed stacking. (c) *μ*-PL spectra of the individual co-crystal. Insets are corresponding bright-field and PL images. (d) Typical bright-field and PL images of a micro-block excited by a 351-nm laser. Reprinted with permission from reference [65]. Copyright 2015 American Chemical Society.

When the single bond between two pyridine groups is replaced by other hydrocarbon bonds, a variety of bipyridyl derivatives are explored in the construction of halogen-bonding supramolecular architectures. Self-assembly of 1,2-bis(4-pyridyl)ethane (B4PyEa) [59,60], 4-(3-(pyridin-4-yl)propyl)pyridine (PyPPy) [61] or 1,2-bis(4-pyridyl)ethylene (B4PyEe) [55,62–65] with 1,4-DITFB has given rise to a 1 : 1 co-crystal, in which bipyridyl derivatives and 1,4-DITFB are linked by intermolecular C–I$\cdots$N halogen-bonding interactions into infinite 1D chains and these chains are connected with each other through C–H$\cdots$F hydrogen-bonding interactions. Thereinto, block-like co-crystal (B4PyEe)$\cdot$(1,4-DITFB) with 2D morphology, stacking in a mixed fashion, was measured to be an insulator and exhibited a unique white-light emission that mainly came from the individual component B4PyEe, indicating that incorporation of the 1,4-DITFB molecule does not change its intrinsic spectroscopic state, which is confirmed by density functional theory (DFT) calculations [65]. Under the excitation of a 351-nm laser, a unique 2D optical waveguide property was demonstrated (Fig. 6).

**Figure 7.**
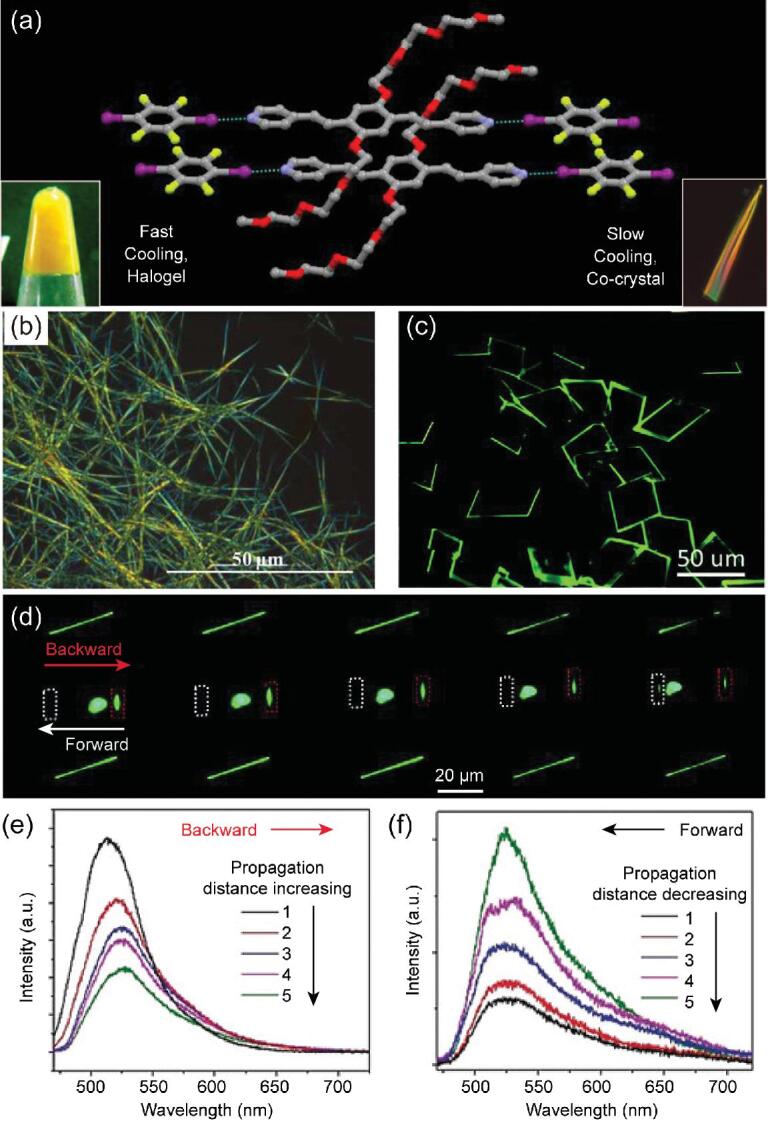
(a) Formation of gel and co-crystal from OPV-2 and 1,4-DITFB due to fast and slow cooling, respectively. (b) Polarized optical microscopic images of the halogels (OPV-2)$\cdot$(1,4-DITFB). (c) The fluorescence microscopy image of co-crystal (OPV-3)$\cdot$(1,4-DITFB) excited with the ultraviolet (UV) band (330–380 nm) from a mercury lamp. (d) The fluorescence microscopy images obtained from an individual 2D (OPV-3)$\cdot$(1,4-DITFB) co-crystal by exciting with a laser beam (λ = 375 nm) at different positions along the [010] direction. (e) and (f) The corresponding spatially resolved PL spectra for the upward and downward direction, respectively. Reprinted with permission from references [66] and [67]. Copyright 2016 American Chemical Society and 2018 Wiley.

Subsequently, *p*-pyridyl-ended oligo *p*-phenylenevinylene (OPV) derivatives (R = *n*-C_4_H_9_, OPV-1; R = (C_2_H_4_O)_3_CH_3_, OPV-2) were employed as the halogen-bonding acceptors [66]. When the hot solutions of OPV derivatives and 1,4-DITFB in equimolar amounts cooled slowly (EtOH for OPV-1; the addition of 20% H_2_O to EtOH for OPV-2), two co-crystals were obtained, forming infinite chains via C–I$\cdots$N halogen bonds. Interestingly, fast cooling of the hot solution for OPV-2 causes fibrous aggregates and eventually supramolecular halogels (Fig. 7a and b). The methoxy substitution (R = CH_3_, OPV-3) generates a 2D organic parallelogram co-crystal that shows an asymmetric optical waveguide (optical-loss coefficients *R*_Backward_ = 0.0346 dB μm^−1^ and *R*_Forward_ = 0.0894 dB μm^−1^), which is ascribed to the unidirectional total internal reflection induced by the anisotropic molecular packing mode (Fig. 7c–f) [67]. The asymmetric photon transport has been exploited as a microscale optical logic gate with multiple in/output channels.

**Figure 8.**
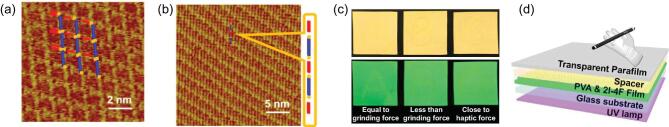
High-resolution scanning tunneling microscopy (STM) images of (*m*-PVA)$\cdot$(1,4-DITFB)_2_ (a) and (*p*-PVA)$\cdot$(1,4-DITFB) (b). Tunneling conditions: *V*_bias_ = 742 mV, *I*_t_ = 460 pA. (c) Practical fluorescence images of the haptic memory device under excitation at 365 nm after touch drawing. (d) Schematic representation of the haptic memory device. Reprinted with permission from reference [68]. Copyright 2017 American Chemical Society.

Recently, two larger conjugated bipyridyl acceptors were exploited by Zhao's group, namely isomeric pyridylvinylanthracenes (*m*-PVA and *p*-PVA) [68]. Assisted by halogen-bonding interactions between PVA and 1,4-DITFB, (*m*-PVA)$\cdot$(1,4-DITFB)_2_ features a porous supramolecular structure (Fig. 8a), whereas (*p*-PVA)$\cdot$(1,4-DITFB) gives a linear assembly (Fig. 8b). The addition of 1,4-DITFB has broadened the fluorescent range of PVA molecules by virtue of the CT effect. The two supramolecular assemblies display remarkable piezochromic behavior with color changes in response to an external stimulus (e.g. pressure or grinding) and can be recovered under mild heating at 60°C, making them promising piezochromic materials for haptic memory (Fig. 8c and d).

When it comes to acetylides, four co-crystals were prepared based on bis(aryl)diacetylenes (4-(2-(pyridin-4-yl)ethynyl)pyridine (PyEPy) [69], 1,4-bis(3-pyridyl)-1,3-butadiyne (B3PyBD), 1,4-bis(3-isoquinolyl)-1,3-butadiyne (B3IQBD) and 1,4-bis(4-isoquinolyl)-1,3-butadiyne (B4IQBD) [70]). In all cases, infinite chains are observed due to C–I⋯N halogen bonds. After the report on the above diacetylene-linked N-heterocycles, an asymmetric ditopic halogen-bonding acceptor, 3-methoxy-1,2-bis(3-pyridyl)ethyne (MeOB3PyE), was discussed by Aakeröy *et al*. [46]. However, halogen-bonding interactions only exist between the more basic nitrogen atom from methoxy-containing pyridine and iodine atoms of 1,4-DITFB owing to its higher electron density, resulting in the discrete trimers.

The introduction of heteroatoms between two pyridine groups does not change the binding site of halogen-bonding acceptors, such as 4-(pyridin-4-ylsulfanyl)pyridine (PySPy) [71] and 4,4'-azopyridine (APy) [64]. The sulfur atom or azo moiety is not engaged in any inter/intramolecular interaction and linear-extended halogen-bonding chains still occur between the pyridine N atom and iodine atom of 1,4-DITFB. In addition, the structural equivalence of the azo (−N = N−) in APy and the ethylene (−C = C−) from B4PyEe mentioned above has been demonstrated by Varughese and co-workers in the co-crystallization process with 1,4-DITFB.

Although similar halogen-bonding chains were observed in the 1 : 1 co-crystals of dipyridyl ureas (1,3-bis((pyridin-2/3/4-yl)methyl)urea (*o/m/p*-BPyMU)) and 1,4-DITFB [72], the intermediate ureas behave differently from the sulfur atom and azo moiety stated above. They participate in the intermolecular N–H$\cdots$O interactions and induce the formation of 1D urea–urea hydrogen-bonding tapes. In regard to polymorphic (*p*-BPyMU)$\cdot$(1,4-DITFB)_2_, nitrogen atoms of pyridine and urea both act as the halogen-bonding acceptors, affording a 2D supramolecular grid structure.

**Figure 9.**
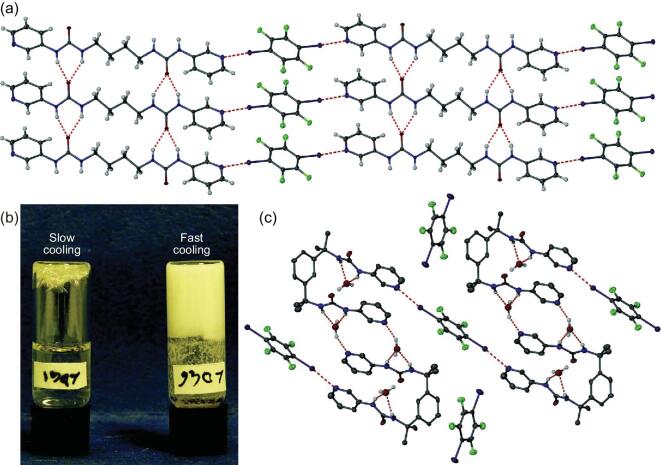
(a) 2D sheet structure through intermolecular interactions in (BPyU-1)$\cdot$(1,4-DITFB). (b) Formation of gel and co-crystal from BPyU-1 and 1,4-DITFB as a result of fast and slow cooling, respectively. (c) Halogen bonding and water inclusion in (BPyU-2)$\cdot$(1,4-DITFB)$\cdot$2H_2_O. Reprinted with permission from reference [33]. Copyright 2013 Nature.

Replacing BPyMU with bis(pyridyl urea) derivatives (BPyU-1 and BPyU-2), two new co-crystals, (BPyU-1)$\cdot$(1,4-DITFB) and (BPyU-2)$\cdot$(1,4-DITFB)$\cdot$2H_2_O, were isolated when hot solutions of BPyU and 1,4-DITFB in methanol–water mixtures cooled slowly [33]. In a manner similar to (*o/m/p*-BPyMU)$\cdot$(1,4-DITFB) above, the former co-crystal with a needle morphology exhibits infinite halogen-bonding chains between 1,4-DITFB and BPyU-1 molecules, with a N$\cdots$I distance of 2.819 Å. Such chains are connected with each other through the N–H$\cdots$O hydrogen bonds among ureas, inducing a 2D sheet structure with urea tapes (Fig. 9a). If the hot solution is allowed to cool rapidly, a supramolecular gel would come into being (Fig. 9b). In the latter case, 1,4-DITFB molecules possess two different environments: one is independent and not involved in any halogen bonds; the other acts as the halogen-bonding bridge between two BPyU-2 molecules, leading to a trimeric assembly that connects adjacent ones into a 1D undulated chain by solvent water via the O–H$\cdots$N hydrogen bonds (Fig. 9c). The absence of urea tapes perhaps explains the relatively weak nature of the gels formed by BPyU-2 and 1,4-DITFB.

As for the system of dipyridyl acetylacetone (1,3-bis(2/3/4-pyridyl)-1,3-propanedione (*o/m/p*-BPyPDONE)), the *β*-diketone group often exists in the form of the enol tautomer described above, producing a six-membered hydrogen-bonding ring via a short O–H$\cdots$O interaction [73]. In (*o*-BPyPDONE)$\cdot$(1,4-DITFB) and (*p*-BPyPDONE)$\cdot$(1,4-DITFB), C–I$\cdots$N halogen bonds guide the formation of 1D zigzag chains that are connected by C–H$\cdots$F and C–H$\cdots$O hydrogen bonds into 3D supramolecular networks. Nevertheless, *m*-BPyPDONE shows a different way: if co-crystallizing with 1,4-DITFB in a 1 : 1 ratio, one oxygen and nitrogen atom in each molecule both interact with the iodine atoms respectively (d_I$\cdots$N_ = 2.895 Å, d_I$\cdots$O_ = 3.072 Å), offering an almost linear 1D chain; if co-crystallizing with 1,4-DITFB in a 2 : 1 ratio, only one nitrogen atom serves as the halogen-bonding acceptor, generating a discrete trimeric motif. In terms of the cyclic carbonyl compounds (4,5-diazafluoren-9-one (DAFONE) and 1,10-phenanthroline-5,6-dione (PDONE)), both the pyridyl N atoms and the carbonyl O atoms are ready to form halogen bonds with I atoms from 1,4-DITFB, leading to infinite 1D assemblies [74].

**Figure 10.**
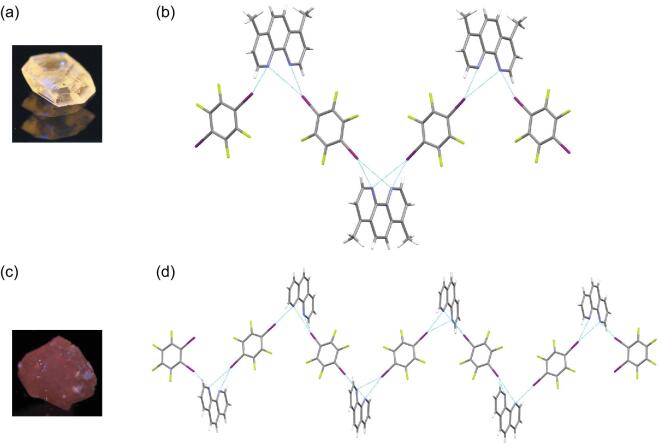
Photos of phosphorescent co-crystals (DMPhen)_3_$\cdot$(1,4-DITFB)_4_ (a) and (1,10-Phen)_6_$\cdot$(1,2-DITFB)_2_$\cdot$(1,4-DITFB)_5_ (c) under UV-365-nm irradiation. Discrete supramolecular assemblies: (b) a heptameric motif in (DMPhen)_3_$\cdot$(1,4-DITFB)_4_ and (d) a 13-molecule finite chain in (1,10-Phen)_6_$\cdot$(1,2-DITFB)_2_$\cdot$(1,4-DITFB)_5_. Reprinted with permission from references [77] and [75]. Copyright 2017 International Union of Crystallography and Elsevier.

With respect to phenanthrolines, discrete trimers are more common based on C–I$\cdots$N halogen-bonding interactions in their co-crystals (4,7-phenanthroline (4,7-Phen) [75], 1,10-phenanthroline (1,10-Phen) [57,75], 2-chloro-1,10-phenanthroline (ClPhen) [76], 4,7-dichloro-1,10-phenanthroline (DClPhen), 4,7-diphenyl-1,10-phenanthroline (DPhPhen) [77]), except one case of 1D chain (1,7-phenanthroline (1,7-Phen) [75]) and heptameric motif (4,7-dimethyl-1,10-phenanthroline (DMPhen) [77]) (Fig. 10a and b). Besides, a 13-molecule finite chain was found in the three-component co-crystal by assembling 1,10-Phen with both 1,4-DITFB and 1,2-DITFB (Fig. 10c and d) [75]. The phosphorescence of all co-crystals is realized by the more efficient spin–orbit coupling by virtue of halogen-bonding interactions between phenanthrolines and 1,4-DITFB, with an obvious change in colors from green to red.

Self-assembly of 1,4-DITFB with the tripyridyl compound, 2,2^′^,2^″^-terpyridine (TPy) [78] or 1,3,5-tris(4-pyridyl(ethenyl))benzene (TPyEB) [79], has led to a halogen-bonding co-crystal with a 2 : 1 molar ratio, crystallizing in the *P*2_1_/*c* space group. In the two co-crystals, only one of three pyridyl groups is halogen bonded to 1,4-DITFB, affording the trimeric adducts.

**Figure 11.**
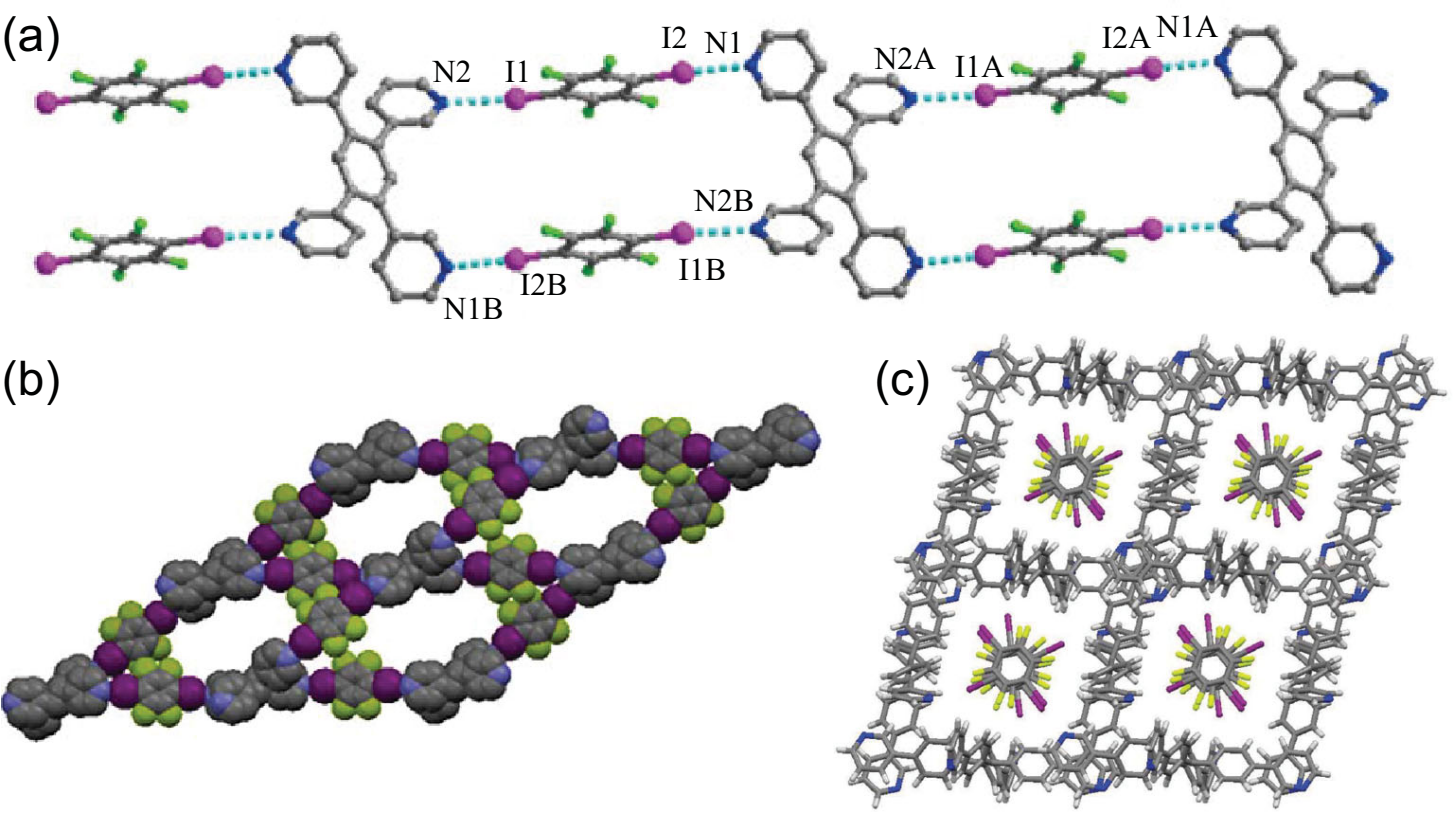
(a) 1D linear double chain containing the square grid network in (TPy3B)$\cdot$(1,4-DITFB)_2_. (b) Schematic representation of the 2D square networks in (TPyCB)$\cdot$(1,4-DITFB)_2_. (c) View of the extended packing down *c* in (TPy3PE)$\cdot$(1,4-DITFB)_2_. Reprinted with permission from references [81], [82] and [80]. Copyright 2015 Springer, 2010 Elsevier and 2013 Royal Society of Chemistry.

When extending this work to the tetrapyridyl system, several co-crystals with interesting halogen-bonding substructures have been published: (i) donor–acceptor dimers: (TPy4PE)$\cdot$(1,4-DITFB) (TPy4PE = 1,1,2,2-tetra(4-(pyridin-4-yl)phenyl)ethene) [80]; (ii) 1D chains: (TPy3B)$\cdot$(1,4-DITFB)_2_ (TPy3B = 1,2,4,5-tetra(pyridin-3-yl)benzene), (TPy4B)$\cdot$(1,4-DITFB) (TPy4B = 1,2,4,5-tetra-(pyridin-4-yl)benzene) [81]; (iii) 2D networks: (TPyCB)$\cdot$(1,4-DITFB)_2_ (TPyCB = tetrakis(4-pyridyl)cyclobutane) [82], (TPyVPM)$\cdot$(1,4-DITFB)_2_ (TPyVPM = tetrakis(4-((*E*)-2-(pyridin-4-yl)vinyl)phenyl)methane) [83]; (iv) interpenetrated 3D networks: (TPy3PE)$\cdot$(1,4-DITFB)_2_ (TPy3PE = 1,1,2,2-tetra(4-(pyridin-3-yl)phenyl)ethene) [80]. X-ray crystallographic studies of these compounds have demonstrated that high-dimensional supramolecular networks are often produced if all N atoms of four pyridyl groups participate in C–I$\cdots$N halogen-bonding interactions; low-dimensional supramolecular assemblies (discrete structures or 1D chains) may appear if they are ‘unsaturated’ in a supramolecular sense (Fig. 11).

**Figure 12.**
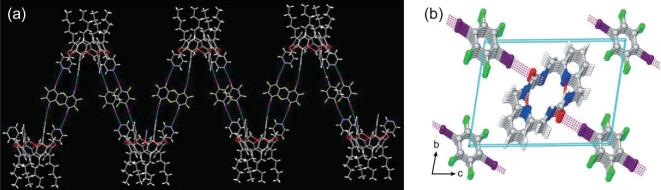
(a) Highly undulated infinite ribbon in (PTPyC)$\cdot$(1,4-DITFB)_2_. (b) Packing diagrams for (BPyBUM)$\cdot$(1,4-DITFB). Reprinted with permission from references [86] and [87]. Copyright 2012 and 2017 Royal Society of Chemistry.

A new class of cyclic compounds with terminal pyridyl groups were chosen as halogen-bonding acceptors by Resnati and co-workers, including 1,3-bis-pyridylmethylcalix[4]arene (BPyMCA) [84], [N_3_P_3_(2,2^′^-dioxybiphenyl)_2_- (4-pyridinoxy)_2_] ((N_3_P_3_)(DOBP)_2_(PyO)_2_) [85] and C-pentyltetra(4-pyridyl)cavitand (PTPyC) [86]. In the case of BPyMCA, only one pyridyl N atom interacts with the iodine atom from 1,4-DITFB through C–I$\cdots$N halogen bonds so that discrete trimeric assemblies emerge. Regarding (N_3_P_3_)(DOBP)_2_(PyO)_2_ and PTPyC, all N atoms of pyridyl groups are engaged in halogen bonds, giving rise to a 1D chain and highly undulated infinite ribbon (Fig. 12a), respectively.

However, the situation is different when pyridyl groups are incorporated into a macrocycle with ureas, such as 1,3-bis((pyridin-2-yl)methyl) bis-urea macrocycle (BPyBUM) [87]. Two O lone pairs are poised on the exterior per macrocycle, which form both C–I$\cdots$O halogen bonds with the iodine atoms from 1,4-DITFB and N–H$\cdots$O hydrogen bonds with the NH group of ureas, leaving the protected pyridine nitrogen atom free to act as the hydrogen-bonding acceptor of the NH group (Fig. 12b).

If halogen-bonding acceptors contain other N-heterocycles besides pyridines, primary intermolecular interactions are more unpredictable, for example, 2,3-bis(pyridin-2-yl)pyrazine (BPyP) [88], 3-(1-methylpyrrolidin-2-yl)pyridine (MPPy) [89], 4-(1*H*-pyrazol-3-yl)pyridine (PPy), 2-(pyridin-4-yl)-1*H*-benzo[*d*] imidazole (PyBI) [46] and imidazo[4,5-*b*]pyridine (IPy) [90]. In the co-crystal of MPPy with 1,4-DITFB, both pyridine and pyrrole N atoms take part in the halogen bonds, offering 1D supramolecular assemblies. The C–I$\cdots$N_pyrrole_ length (3.015 Å) is much longer than the C–I$\cdots$N_pyridine_ distance (2.873 Å), suggesting that the pyridine nitrogen is the preferred binding site. In other cases, the I atoms of 1,4-DITFB only interact with pyridine nitrogen (PyBI) or other heterocyclic nitrogen (BPyP, PPy, IPy), generating discrete halogen-bonding dimeric or trimeric motifs.

**Figure 13.**
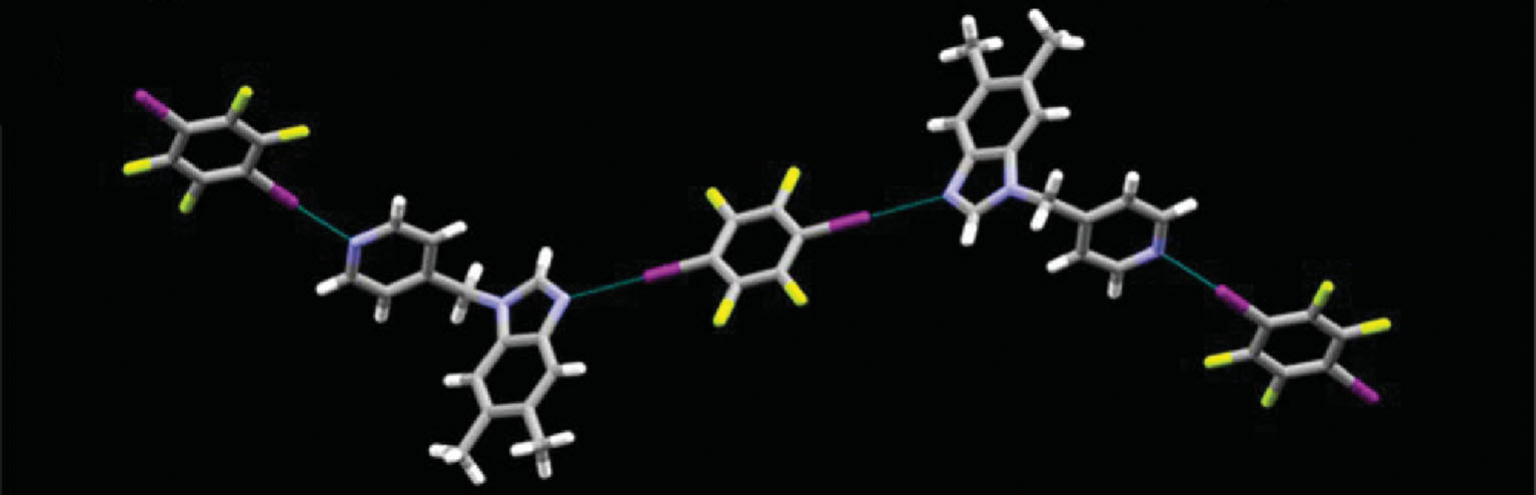
Halogen-bonding interactions in (DMPy4MBI)_2_$\cdot$(1,4-DITFB)_3_. Reprinted with permission from reference [91]. Copyright 2016 American Chemical Society.

With a view to predicting halogen-bonding selectivity effectively, Aakeröy *et al.* performed systematic co-crystallizations on pyridine-containing imidazoles with two different acceptor sites, i.e. 1-(pyridin-3-ylmethyl)-1*H*-benzo[*d*]imidazole (Py3MBI), 1-(pyridin-4-ylmethyl)-1*H*-benzo[*d*]imidazole (Py4MBI), 5,6-dimethyl-1-(pyridin-3- ylmethyl)-1*H*-benzo[*d*]imidazole (DMPy3MBI), 5,6-dimethyl-1-(pyridin-4- ylmethyl)-1*H*-benzo[*d*]imidazole (DMPy4MBI), 3-((1*H*-imidazol-1 -yl)methyl)pyridine (3IMPy), 4-((1*H*-imidazol-1-yl)methyl)pyridine (4IMPy), 4-((2-phenyl-1*H*-imidazol-1-yl)methyl)pyridine (PIMPy) [91]. Electrostatic potential calculations indicate that, if the potential-energy difference between two binding sites is not large enough (<75 kJ/mol), halogen-bonding selectivity will disappear and both potential acceptor sites will simultaneously participate in halogen-bonding interactions. As with every situation here except PIMPy, both pyridine and imidazole N atoms are halogen bonded to 1,4-DITFB via C–I$\cdots$N interactions (Fig. 13).

##### Other aromatic nitrogen-containing heterocycles.

**Figure 14.**
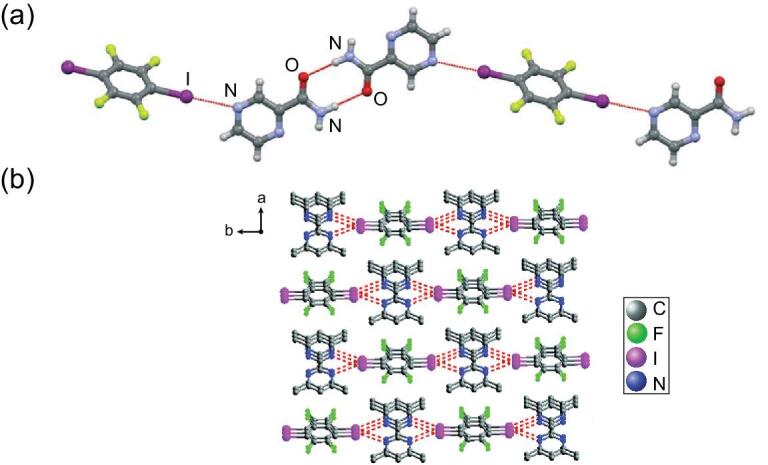
(a) Intermolecular interactions in (Pyrazinamide)_2_$\cdot$(1,4-DITFB). (b) Halogen-bonding interactions in (TMBP)$\cdot$(1,4-DITFB). Reprinted with permission from references [94] and [74]. Copyright 2017 Royal Society of Chemistry and 2011 American Chemical Society.

On top of pyridine derivatives, other aromatic nitrogen-containing heterocycles can also work as the halogen-bonding acceptors such as pyrazine compounds (phenazine [92], 2-aminopyrazine (AP), 2-amino-5-bromopyrazine (ABP), 2-amino-3,5-dibromopyrazine (ADBP) [93], pyrazinamide [94], *N-*(pyrazin-2-yl)isobutyramide (PIBA) [95]). Just like acridine with one binding site [92], only one nitrogen atom in these pyrazine compounds interacts with the iodine atom from 1,4-DITFB, resulting in termolecular structures that connect to each other by N–H$\cdots$N/O or C–H$\cdots$N hydrogen-bonding interactions into a 1D motif (Fig. 14a). Nevertheless, all nitrogen atoms of pyrazine are engaged in the C–I$\cdots$N halogen bonds [41], forming infinite 1D chains, as are the cases for 2,3,5,6-tetramethylpyrazine (TMP) [57], quinoxaline [41] and 4,4^′^,6,6^′^-tetramethyl-2,2^′^-bipyrimidine (TMBP) (Fig. 14b) [74].

**Figure 15.**
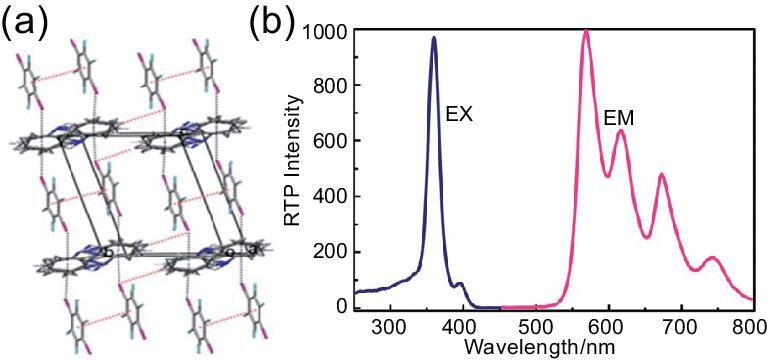
(a) Crystal packing in the co-crystal (Carbazole)$\cdot$(1,4-DITFB)_2_ formed by C–I$\cdots$π interactions and π$\cdots$π stacking. (b) Phosphorescent excitation and emission spectra of the co-crystal. Reprinted with permission from reference [96]. Copyright 2012 Royal Society of Chemistry.

After the report on the above pyrazine compounds, interest in azoles has been going on. Carbazole became the first research object and a triclinic co-crystal with 1,4-DITFB was synthesized in a 1 : 2 molar ratio, driven primarily by C–I$\cdots$π intermolecular interactions and synergistically by C–H$\cdots$F/I hydrogen bonds, π$\cdots$π stacking as well as F$\cdots$F contacts (Fig. 15a) [96]. Introduction of 1,4-DITFB as the heavy-atom disturber affects the luminescent characteristic of the carbazole molecule and induces a strong phosphorescence emission by spin–orbit coupling (Fig. 15b). It is probably the first report on a phosphorescent co-crystal assembled by weak C–I$\cdots$π interaction. Steric effects of *N*-substituted groups (*N*-methyl or *N*-ethyl) on the carbazole backbone result in the different π-hole$\cdots$π and C–I$\cdots$π interaction modes [97] and engender the blue- or red-shift phosphorescence spectra compared with the co-crystal (Carbazole)$\cdot$(1,4-DITFB)_2_.

Thereafter, 2,5-diphenyloxazole (DPO), a well-known UV fluorescent material, was utilized to co-crystallize with 1,4-DITFB through C–I$\cdots$N halogen bonds and explore novel photoelectric properties [98]. The halogen-bonding trimeric complex, (DPO)$\cdot$(1,4-DITFB)_2_, shows UV/blue polarized emission, the second harmonic generation effect and reversible mechanochromic fluorescence (MCF) properties. Likewise, DPO-based co-crystals can be constructed based on other co-formers (1,4-dibromotetrafluorobenzene, tetrafluoroterephthalic acid and so on), presenting tunable fluorescence properties in the UV/blue region [99]. Upon the formation of co-crystals, their dielectric constants have a significant increase due to the introduction of the hydrogen- and/or halogen-bonding interactions.

**Figure 16.**
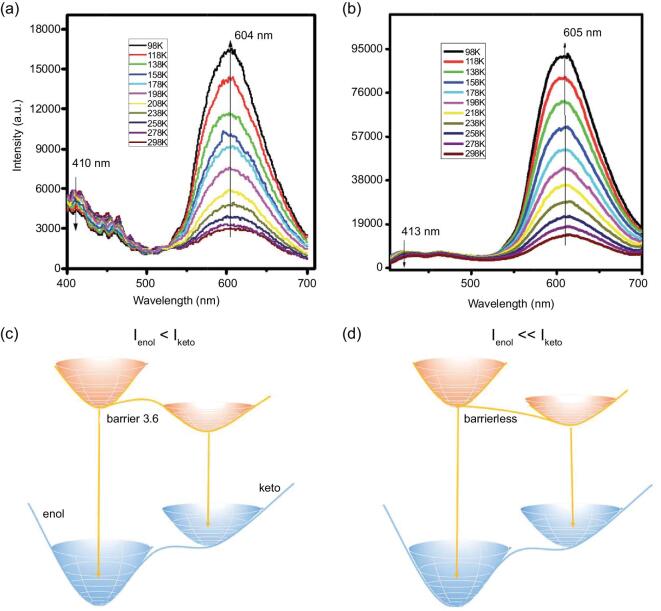
PL emission of HMPBTA (a) and its co-crystal (HMPBTA)$\cdot$(1,4-DITFB)_2_ (b) at different temperatures. Schematic energy levels of the S_1_ enol and keto species of HMPBTA (c) and its co-crystal (HMPBTA)$\cdot$(1,4-DITFB)_2_ (d). Reprinted with permission from reference [101]. Copyright 2017 Royal Society of Chemistry.

The substitution of the triazole ([1,2,3] triazolo[1,5-*α*]quinoline (TAQ) [100] or 2-(2^′^-hydroxy-5^′^-methyl-phenyl)-benzotriazole (HMPBTA) [101]) for DPO gives rise to two new co-crystals with similar halogen-bonding trimeric structures, where only one nitrogen atom is halogen bonded to 1,4-DITFB. In the latter case, the formation of co-crystal (HMPBTA)$\cdot$(1,4-DITFB)_2_ leads to enhanced excited-state intramolecular proton transfer (ESIPT) emission compared with the pristine HMPBTA (Fig. 16). The emission band of ∼413 nm is assigned to the fluorescence emission of its S_1_ enol while the one at ∼605 nm stems from the emission of the S_1_ keto species. The ESIPT process is computed to be barrierless, so the S_1_ keto species are populated much more than the enol, which explains why the emission band of ∼413 nm nearly disappears.

**Figure 17.**
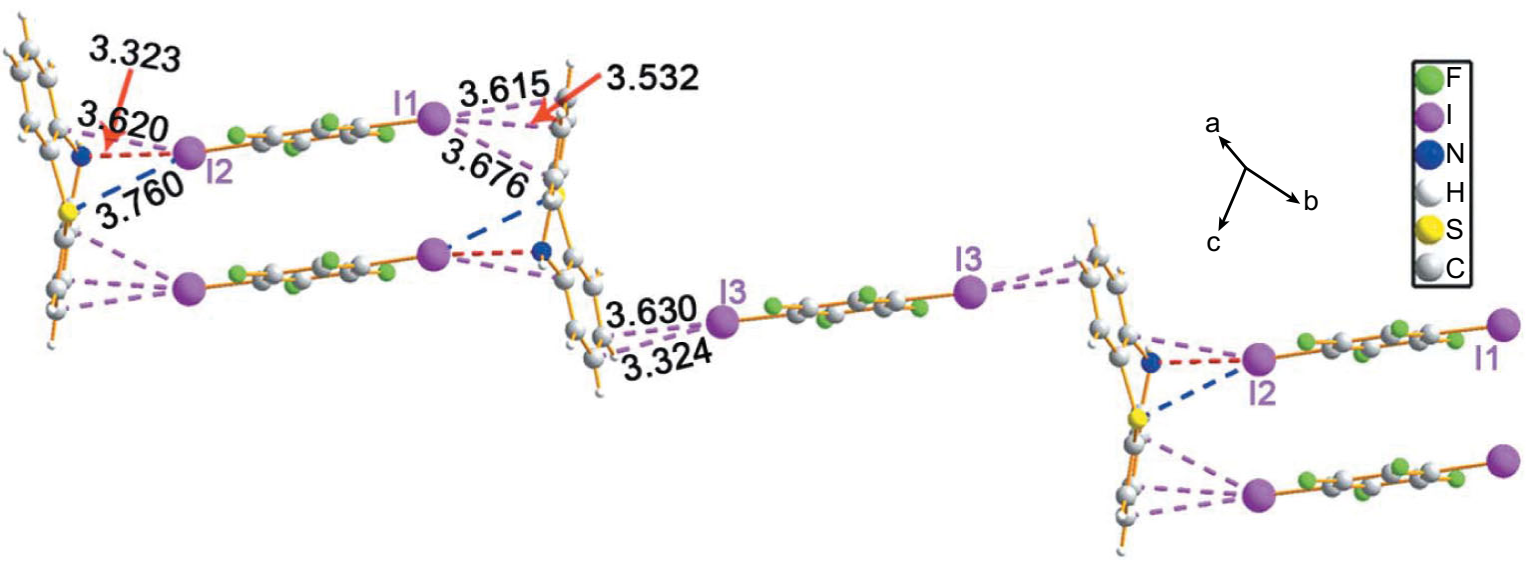
1D halogen-bonding chain through C–I$\cdots$N/S/π interactions in (Phenothiazine)_2_$\cdot$(1,4-DITFB)_3_. Reprinted with permission from reference [102]. Copyright 2017 International Union of Crystallography.

In Jin's group, a new co-crystal was successfully assembled by the butterfly-shaped non-planar phenothiazine and 1,4-DITFB [102]. Single-crystal X-ray diffraction analysis reveals that a 1D chain exists in the co-crystal with the *A*$\cdots$*2D*$\cdots$*A*$\cdots$*D*$\cdots$ (A = acceptor, D = donor) arrangement owing to C–I$\cdots$N/S/π halogen bonds, wherein the iodine atom from 1,4-DITFB can participate in bifurcated or trifurcated interactions (Fig. 17). Study of the luminescent property indicates that the complex emits relatively strong delayed fluorescence with a small stokes shift and weak phosphorescence, obviously different from those of co-crystals constructed by rigid planar molecules such as monoazaphenanthrenes [42] and carbazole [96]. The phenothiazine molecule has a large torsion angle of 150.94° between two wings and the non-planarity makes the phosphorescent radiative transition process decrease, thus producing weak phosphorescence.

#### Aliphatic nitrogen-containing heterocycles

Attempts were also made to use aliphatic nitrogen-containing heterocycles (e.g. hexamethylenetetramine (HMTA) [55] and 1,4-diazabicyclo[2.2.2]octane (DABCO) [92,103,104]) as the halogen-bonding acceptors of 1,4-DITFB. Two co-crystals (HMTA)$\cdot$(1,4-DITFB) and (DABCO)$\cdot$(1,4-DITFB) were prepared by solution methods, where the intermolecular C–I$\cdots$N halogen bonds direct the formation of 1D zigzag chains in the former but linear chains in the latter. Moreover, three isostructural co-crystals with a 1 : 1 molar ratio were synthesized by mechanochemical methods (such as grinding) based on six-membered heterocyclic compounds (piperazine, morpholine and thiomorpholine (Tmor)) [103]. Similar zigzag halogen-bonding chains are obtained by virtue of C–I$\cdots$N/O/S halogen bonds.

**Figure 18.**
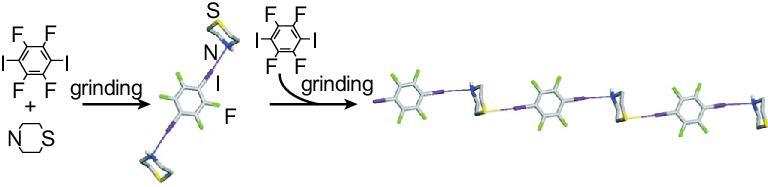
Stepwise mechanism for the mechanochemical synthesis of co-crystals from Tmor and 1,4-DITFB. Reprinted with permission from reference [105]. Copyright 2008 American Chemical Society.

In order to explore the mechanism of mechanochemical co-crystallization, the co-crystal of Tmor with 1,4-DITFB was selected as the model system by Jones *et al.* [105]. Grinding 1 : 1 reactants for 30 min provided infinite zigzag chains of (Tmor)$\cdot$(1,4-DITFB), whereas a shorter time of grinding (e.g. 4 min) offered not merely halogen-bonding chains, but also trimeric assemblies of (Tmor)_2_$\cdot$(1,4-DITFB) that is isostructural with (Piperidine)_2_$\cdot$(1,4-DITFB). The appearance of discrete assemblies as the intermediate reaction can be explained by the hierarchy of halogen bonds, namely the nitrogen atom as the better acceptor is the preferred binding site, and stronger C–I$\cdots$N halogen bonds initially drive the formation of trimeric (Tmor)_2_$\cdot$(1,4-DITFB); further grinding leads to polymerization of the assemblies via weaker C–I$\cdots$S bonds, forming (Tmor)$\cdot$(1,4-DITFB). Consequently, a stepwise mechanism for the mechanochemical synthesis of halogen-bonding co-crystals has been proposed and demonstrates the competition between supramolecular interactions (Fig. 18).

**Figure 19.**
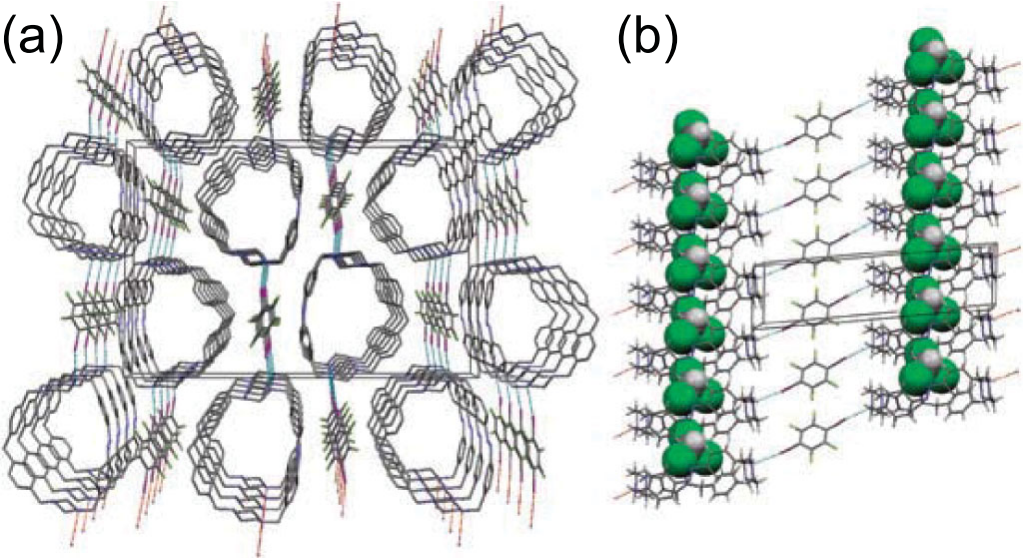
(a) 1D halogen-bonding motifs in (PC-2)$\cdot$(1,4-DITFB). (b) The inclusion of chloroform molecules into well-defined tubular structures of PC-2 in the co-crystal. Reprinted with permission from reference [106]. Copyright 2009 Royal Society of Chemistry.

Additionally, piperazine cyclophanes (PC-1 and PC-2) with a rigid macrocyclic skeleton have been employed in the deliberate design of binary co-crystals by Rissanen's group [106]. During their co-crystallization process with 1,4-DITFB, halogen bonds C–I$\cdots$N guide the self-assembly of piperazine cyclophanes with 1,4-DITFB into termolecular and 1D motifs separately, affording well-defined tubular structures with solvent chloroform inclusion (Fig. 19). Thereafter, the behaviors of amino piperazine cyclophanes (TAPC-1 and TAPC-2) were investigated in their co-crystals (TAPC-1)$\cdot$(1,4-DITFB) and (TAPC-2)_2_$\cdot$(1,4-DITFB) [107], in which the amino groups are hydrogen bonded to the adjacent piperazine nitrogen atoms to generate intramolecular N–H$\cdots$N interactions, and no solvent molecules were encapsulated. In the former case, two piperazine N atoms in each TAPC-1 molecule interact with iodine atoms from 1,4-DITFB, giving rise to 1D halogen-bonding assemblies analogous to those in the co-crystal of PC-2. As for (TAPC-2)_2_$\cdot$(1,4-DITFB), TAPC-2 folds into a very compact shell-like conformation because of the intramolecular N–H$\cdots$N hydrogen-bonding interactions stated above, and hence one of the amino N atoms with the lower nucleophilicity serves as the halogen-bonding acceptor, leading to weak intermolecular C–I$\cdots$N halogen-bonding interactions (d_I$\cdots$N _= 2.946 or 2.993 Å, ∠C–I$\cdots$N = 175.63 or 169.18°).

#### Acyclic nitrogen-containing compounds

With the aim of establishing the halogen-bonding hierarchy, Cinčić and co-workers have performed co-crystallization experiments on a series of aromatic amines with various functional groups (3-aminobenzonitrile (ABN), 4-nitroaniline (NA), 4-aminoacetophenone (AAP), 4-aminobenzophenone (ABP) [43] and lidocaine [94]). Single-crystal X-ray diffraction studies of their co-crystals with 1,4-DITFB show that the cyano nitrogen atom is a poorer halogen-bonding acceptor, whereas the nitro or carbonyl oxygen atom is a better one compared with the amino nitrogen atom. However, in the co-crystal (ABP)_2_$\cdot$(1,4-DITFB), both the amino nitrogen and carbonyl oxygen atoms participate in the formation of hydrogen bonds rather than halogen bonds, leaving the π-system of the aromatic amine as the halogen-bonding acceptor (d_I$\cdots$π_ = 3.46 Å, ∠C–I$\cdots$π = 167°). In addition to the aromatic amines discussed above, two tertiary amines (*N,N,N^′^,N^′^*-tetramethyl-1,4-phenylenediamine (TMPDA) and bis[4-(*N,N*-dimethylaminophenyl)]methane (BDMAPM)) are exploited to co-crystallize with 1,4-DITFB, providing infinite chains via C–I$\cdots$N halogen bonds [108].

**Figure 20.**
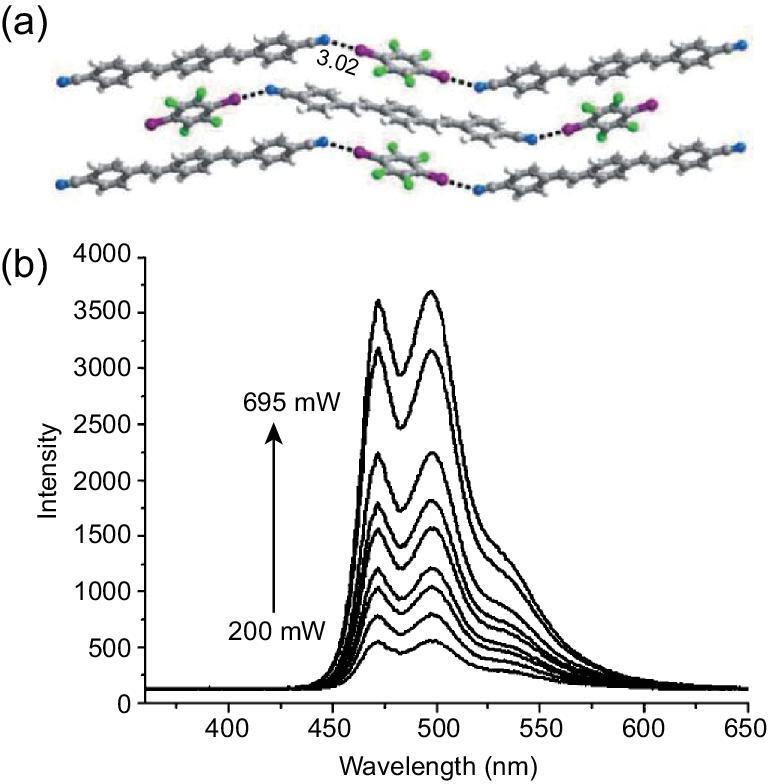
(a) 1D halogen-bonding chain in the co-crystal (BCSB)$\cdot$(1,4-DITFB). (b) Two-photon fluorescence spectra of the co-crystal excited by 800-nm laser under different pump intensities. Reprinted with permission from reference [110]. Copyright 2011 Wiley.

The cyano group can be engaged in the formation of halogen-bonding interactions as well when no other functional groups exist in the compound, for instance, 2,3,5,6-tetramethylterephthalonitrile (TMTPN) [109], 1,4-bis(4-cyanostyryl)benzene (BCSB) [110,111] and 1-(2-cyanostyryl)-4-(4-cyanostyryl)benzene (CSCSB) [112]. In the co-crystal (BCSB)$\cdot$(1,4-DITFB) with 1D halogen-bonding chain that was synthesized by the liquid-assisted grinding method [110], the introduction of 1,4-DITFB changed the stacking mode of chromophore BCSB and enlarged the separation between the BCSB molecules so as to reduce the degree of molecular aggregation, inducing a strong blue shift by 64 nm in contrast with pure BCSB. Upon excitation by an 800-nm laser, the co-crystal exhibits strong two-photon luminescence with two main narrow peaks at 470 and 497 nm (Fig. 20). Compared with the above macroscopic co-crystal, the nanosized counterpart obtained by an ultrasound-assisted crystallization method presents different photoemission properties such as one-/two-phonon emission and fluorescence lifetime, which gives new insights into the size-dependent luminescence effects of multicomponent organic nanocrystals [113]. Recently, the microcrystalline form of (BCSB)$\cdot$(1,4-DITFB), sky-blue-emissive microwire, has been reported [111]. In contrast with the pure BCSB organic microcrystal, its radiative decay (*k*_r_) rate is enhanced from 0.04 to 0.12 ns^−1^ and the fluorescence lifetime goes from 14.0 to 0.9 ns. With regard to the system of its isomer CSCSB, a tetramolecular ring-like structure was observed between CSCSB and 1,4-DITFB molecules via C–I$\cdots$C≇N halogen bonds [112]. The two-component assemblies displayed a slight red shift (from 451 to 462 nm) and photochromic fluorescence.

### N-oxides as the halogen-bonding acceptors

Heteroaromatic N-oxides can serve as effective electron donors towards perfluorocarbon iodides, such as 1,1,3,3-tetramethylisoindolin-2-yloxyl (TMIO) [114]. The intermolecular N–O$\cdots$I–C interactions connect TMIO and 1,4-DITFB into a discrete trimer, demonstrated by thermal analysis and vibrational spectroscopy (Raman and IR), and confirmed by DFT calculations. Electron paramagnetic resonance spectroscopy of TMIO in solution indicates that the halogen bond stabilizes the ionic resonance structure of the nitroxide radical and then leads to an increase in the electron density at the nitroxide nitrogen nucleus. However, if the N-oxide features other potential acceptor sites, the main intermolecular interactions can be complicated and not easy to predict. Taking 4-phenyl-2,2,5,5-tetramethyl-3-imidazolin-1-yloxyl radical (PTMIO) for example [115], imidazole nitrogen is engaged in the C–I$\cdots$N halogen bond instead of the nitroxide radical that forms a weak N–O$\cdots$H–C hydrogen bond.

**Figure 21.**
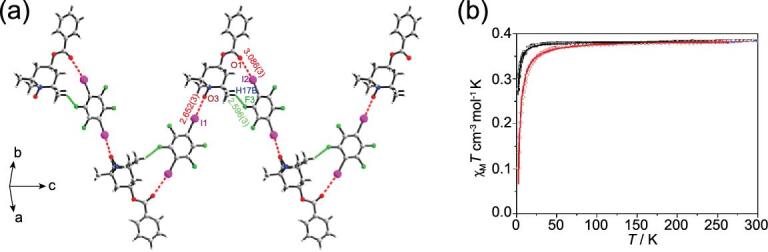
(a) 1D zigzag chain in (BTMPO)$\cdot$(1,4-DITFB). (b) Plots of *χ*_M_*T* vs *T* together with the best-fitting result for BTMPO (black line) and (BTMPO)$\cdot$(1,4-DITFB) (red line). Reprinted with permission from reference [116]. Copyright 2013 American Chemical Society.

As far as the 4-amino-2,2,6,6-tetramethyl(piperidin-1-yloxyl) (ATMPO) [115] or 4-benzo-yloxy-2,2,6,6-tetramethylpiperidine-1-oxyl (BTMPO) [116] free radical is concerned, nitroxide along with amidogen or carbonyl both conduct as the halogen-bonding acceptor of 1,4-DITFB, providing 1D zigzag chains. Thereinto, magnetic study of (BTMPO)$\cdot$(1,4-DITFB) reveals a stronger antiferromagnetic interaction (*J* = −1.65 cm^−1^) than the pure BTMPO (*J* = −0.45 cm^−1^) in spite of the existence of a slightly longer N–O$\cdots$O–N distance (6.308 vs. 6.128 Å), suggesting that the halogen bond is able to transmit spin polarization and favor magnetic interactions, which is further backed by calculations (Fig. 21).

Moreover, similar linear halogen-bonding chains were found in the co-crystals between dioxides (4,4^′^-dipyridyl-N,N^′^-dioxide (DPyDO) [56,63,117], (2-phenyl)-4,4,5,5-tetramethylimidazolin-1-oxyl-3-oxide (PTMIOO) [118]) and 1,4-DITFB. In comparison with (BTMPO)$\cdot$(1,4-DITFB) stated above, the co-crystal (PTMIOO)$\cdot$(1,4-DITFB) shows much weaker antiferromagnetic coupling despite the presence of shorter distances between the neighboring PTMIOO radicals (5.127 and 5.466 Å). It seems unusual and might be related to the alignment of the organic radicals as well as the degree of contribution of the halogen-bonding interactions between the radicals and 1,4-DITFB to the magnetic properties.

**Figure 22.**
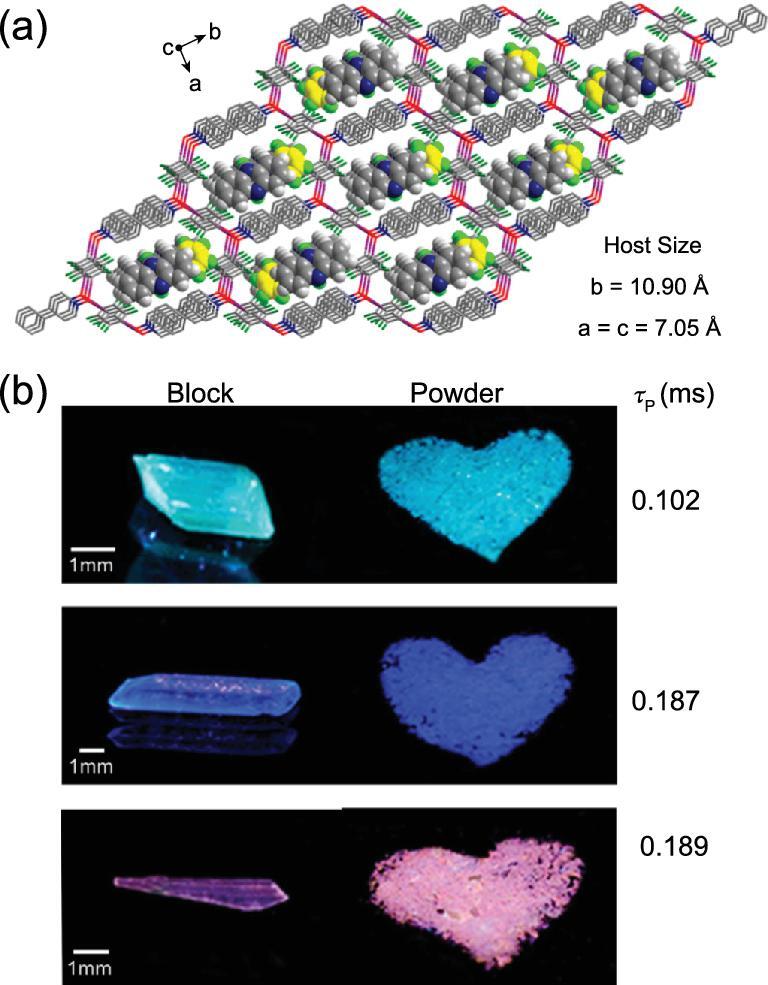
(a) Extracted hexagonal-channel structures of the co-crystal [(PPyNO)$\cdot$(1,4-DITFB)]$\cdot$(Phenazine) as an example of host–guest systems. (b) Photographs of three host–guest co-crystals assembled by PPyNO, 1,4-DITFB and suitable guest molecules (phenazine, acridine, 2,2^′^-BPy) under UV-365 irradiation. Reprinted with permission from reference [119]. Copyright 2018 American Chemical Society.

Recently, pure organic host–guest co-crystals were assembled by 4-phenylpyridine N-oxide (PPyNO) and 1,4-DITFB under the mediation of linear guest molecules (phenazine, acridine and 2,2^′^-BPy) [119]. Robust bifurcated C–I$\cdots$^−^O–N^+^ halogen-bonding interactions between PPyNO and 1,4-DITFB facilitate the formation of 1D zigzag chains that are crossed and interlinked together by C–H$\cdots$F hydrogen-bonding interactions and π−hole$\cdots$π bonds to produce the hexagonal host channels (Fig. 22a). The guest molecules reside in the host channel through π$\cdots$π stacking and interact with the host via C–H$\cdots$π interactions to stabilize the host–guest structure. The host–guest systems show different photoluminescence colors (bright cyan, sapphire blue and pink, respectively) at room temperature, mainly originating from guest molecules (Fig. 22b).

### Chalcogenides as the halogen-bonding acceptors

**Figure 23.**
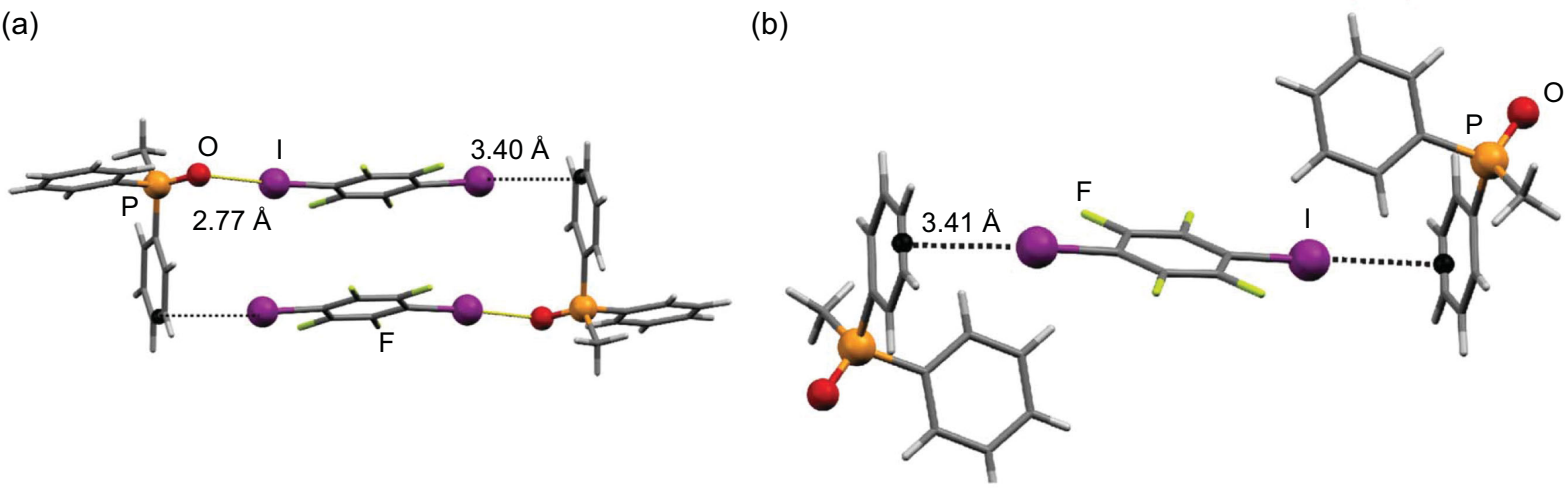
C–I$\cdots$O = P halogen bonds and/or C–I$\cdots$π contacts in (MDPPO)$\cdot$(1,4-DITFB) (a) and (MDPPO)_2_$\cdot$(1,4-DITFB) (b). Reprinted with permission from reference [120]. Copyright 2012 Royal Society of Chemistry.

In addition to nitrogen-containing compounds, chalcogenides are another large category of halogen-bonding acceptors. In recent years, the potential of chalcogenides containing Y=O (Y=C, P, S) as halogen-bonding acceptors has attracted much attention. In terms of phosphine oxides, co-crystallization experiments by mechanochemical methods readily yield polymorphs. For instance, two co-crystals, (MDPPO)$\cdot$(1,4-DITFB) and (MDPPO)_2_$\cdot$(1,4-DITFB) (methyldiphenylphosphine oxide (MDPPO)), were obtained by changing the relative quantities of the starting reactants in a liquid-assisted grinding process [120,121]. In the former case, intermolecular C–I$\cdots$O = P halogen bonds and C–I$\cdots$π contacts direct the formation of tetramolecular fragments, whereas, in the latter, the co-crystal oxygen atom is involved in the C–H$\cdots$O hydrogen-bonding interaction, leaving the π-system of the phenyl ring as the halogen-bonding acceptor (Fig. 23). Here, the competition between hydrogen and halogen bonds gives rise to stoichiometric variations.

Based on triphenylphosphine oxide (TPPO), three co-crystals with different stoichiometric ratios (1 : 1, 1 : 2 and 6 : 5) have been reported, in which C–I$\cdots$O = P halogen bonds are the main driving forces [121–123]. In (TPPO)$\cdot$(1,4-DITFB)_2_ [121], ^17^O and ^31^P solid-state nuclear magnetic resonance spectroscopies indicate the presence of halogen-bonding interactions. The ^17^O quadrupolar coupling constant, ^31^P isotropic chemical shift as well as the asymmetry parameter increase and the *J*(^31^P, ^17^O) coupling constant decreases, compared to the starting material TPPO. Gauge-including projector-augmented wave density functional theory (GIPAW DFT) calculations are in good accordance with experimental results. The natural localized molecular orbital analysis reveals that the three main contributions to *J*(^31^P, ^17^O) coupling are the oxygen core orbital, the oxygen lone-pair orbital and the P = O bonding orbital; the contribution of the oxygen p*_z_* lone-pair orbital to the ^17^O quadrupolar coupling constant displays a linear relationship with the strength of the halogen bond. Afterwards, kinetic study of the co-crystallization process without milling of (TPPO)$\cdot$(1,4-DITFB)_2_ and (TPPO)_6_$\cdot$(1,4-DITFB)_5_ was carried out by *in situ*^31^P solid-state nuclear magnetic resonance (NMR) spectroscopy [123], which makes it clear that the reaction mechanism is primarily diffusion-controlled. This offers an opportunity for real-time monitoring of mechanochemical processes.

When it comes to sulfur oxides, several co-crystals were synthetized based on sulfoxides (dimethyl sulfoxide (DMSO) [124], diphenyl sulfoxide (DPS), phenyl 4-tolyl sulfoxide (PTS), phenyl 2-methoxyphenyl sulfoxide (PMPS), di(2-phenyl)ethyl sulfoxide (DPES) and thianthrene 5-oxide (TAO) [125]). X-ray crystallographic analysis shows that the C–I$\cdots$O=S halogen bond was the dominant interaction in all cases, with a distance range of 2.815–3.096 Å, suggesting that it is a robust supramolecular synthon. In most cases, the oxygen atoms were bifurcated, either involved in two C–I$\cdots$O=S halogen bonds or one C–H$\cdots$O=S hydrogen bond and one C–I$\cdots$O=S halogen bond; when it was not bifurcated, C–I$\cdots$π interactions were present. Concerning sulfinamides (e.g. 4-tolylsulfinamide (TSA)) [126], the oxygen atom serves as both the halogen-bonding and hydrogen-bonding acceptor, leading to the N–H$\cdots$O hydrogen bond and C–I$\cdots$O halogen bond that codetermine the overall supramolecular architecture. A similar situation occurred in the oxygen atom from amides (e.g. acetamide and *N*-methylbenzamide (MBA)) [127] or the sulfur atom from thioamide (e.g. thiourea) [128].

In contrast with the P=O and S=O functional groups, the carbonyl oxygen atom is more commonly used as the halogen-bonding acceptor. The reaction of the diketone compound (cyclohexa-2,5-diene-1,4-dione (CDD) [129] or 1,3-bis(2^′^-caprolactam-1^′^-yl)but-1-ene (BCBE) [130]) with 1,4-DITFB yielded a new co-crystal with extended halogen-bonding chains. A similar polymeric motif was found in the co-crystal of 4,4^′^-bis(dimethylamino)-benzophenone (BDMABP) due to the oxygen atom in a bifurcated manner [131], though the monoketone compound readily generates discrete halogen-bonding assemblies such as lidocaine [94].

For the purpose of testing the potential of the hydroxyl and methoxy group to form halogen bonds, Cinčić *et al.* prepared a series of co-crystals of o-vanillin imine ((*E*)-2-(2-hydroxy-3-methoxybenzylideneamino)naphthalene (2HMBAN), (*E*)-1-(2-hydroxy-3-methoxybenzylideneamino)naphthalene (1HMBAN), (*E*)-1-(2-hydroxy-3-methoxybenzylideneamino)adamantane (HMBAA), (*E*)-1-(4-(2-hydroxy-3-methoxybenzylideneamino)phenyl)ethanone (4HMBAPE), (*E*)-1-(3-(2-hydroxy-3-methoxybenzylideneamino) phenyl) ethanone (3HMBAPE) and (*E*)-4-(2-hydroxy-3-methoxybenzylideneamino)benzonitrile (HMBAB)) [132,133]. In all cases, the imine nitrogen participates in the formation of strong O–H$\cdots$N intramolecular hydrogen-bonding interaction with the hydroxyl group. The hydroxyl or/and methoxy oxygen atom can be halogen bonded to the iodine atom from 1,4-DITFB for the first three co-crystals, but if another functional group exists (e.g. carbonyl or cyano group), that would become the preferred binding site. Actually, as early as 2008, their research team also chose cyclic chalcogenides (1,4-dioxane, 1,4-dithiane and 1,4-thioxane) to react with 1,4-DITFB, producing 1D single chains or ladder-like chains through C–I$\cdots$O/S halogen-bonding interactions [103,122].

**Figure 24.**
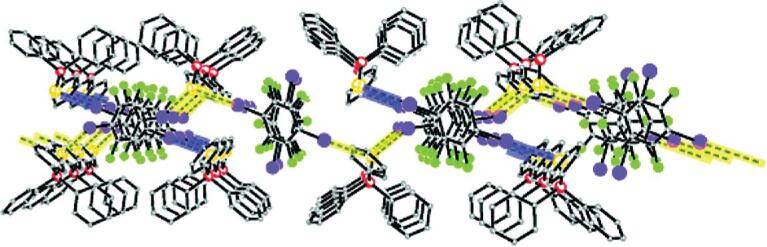
Layer of interwoven chains and termolecular adducts for (TPPS)$\cdot$(1,4-DITFB). Dashed lines in yellow and blue highlight the infinite chains and termolecular adducts, respectively. Reprinted with permission from reference [134]. Copyright 2012 American Chemical Society.

Impressively, a heavier congener of the oxygen atom, selenium, can behave as an excellent halogen-bonding acceptor of organic iodides. In the co-crystal of triphenylphosphine selenide (TPPS) with 1,4-DITFB [134], both monotopic and ditopic selenium atoms were observed, affording termolecular adducts and zigzag chains through C–I$\cdots$Se halogen bonds, respectively, which are interwoven with each other (Fig. 24).

### Aromatic hydrocarbons as the halogen-bonding acceptors

**Figure 25.**
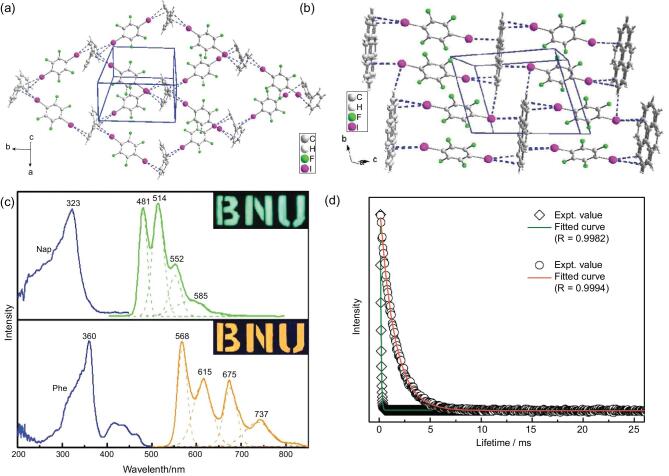
Structures of the co-crystals (Biphenyl)$\cdot$(1,4-DITFB)_2_ (a) and (Phenanthrene)$\cdot$(1,4-DITFB)_2_ (b). (c) Phosphorescent excitation (blue lines) and emission spectra of co-crystals (green line for (Naphthalene)$\cdot$(1,4-DITFB)_2_; orange line for (Phenanthrene)$\cdot$(1,4-DITFB)_2_). (d) Phosphorescence-decay curves of (Naphthalene)$\cdot$(1,4-DITFB)_2_ (green line) and (Phenanthrene)$\cdot$(1,4-DITFB)_2_ (red line). Reprinted with permission from reference [135]. Copyright 2012 Royal Society of Chemistry.

The π-conjugated systems of aromatic hydrocarbons show the ability to offer rich electrons to 1,4-DITFB although no typical functional groups are included. The π-type halogen-bonding acceptors (e.g. biphenyl, naphthalene and phenanthrene) were employed to construct a new family of co-crystals by Jin's group [135]. Each side of the aromatic hydrocarbon connects with two 1,4-DITFB molecules via C–I$\cdots$π contacts (d_I$\cdots$π_ = 3.566, 3.484 and 3.434 Å, respectively), giving rise to a 2D rhombus network structure for (Biphenyl)$\cdot$(1,4-DITFB)_2_ owing to the rotation of the single bond between two benzene rings (Fig. 25a), but 1D grid-like chains for (Naphthalene)$\cdot$(1,4-DITFB)_2_ and (Phenanthrene)$\cdot$(1,4-DITFB)_2_ (Fig. 25b). Notably, the latter two co-crystals display strong phosphorescence ascribed to the spin–orbit coupling of iodine atoms and their decay has a mono-exponential phosphorescence-decay property with lifetimes of 0.067 and 1.449 ms, respectively (Fig. 25c and d).

**Figure 26.**
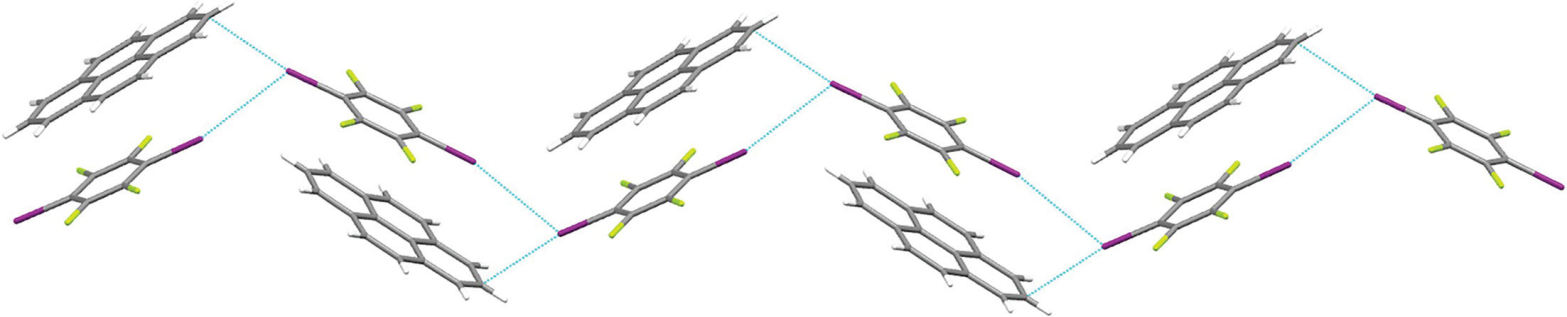
Halogen-bonding chain in (Pyrene)$\cdot$(1,4-DITFB) [136].

However, in the co-crystallization process of pyrene with 1,4-DITFB [136], the main driving forces are π$\cdots$π stacking and I$\cdots$I interactions though weak C–I$\cdots$π contacts occur, presumably as a result of the pyrene molecule with a larger π-conjugation that is prone to stack via π$\cdots$π interactions. X-ray crystallographic analysis reveals that each pyrene molecule alternates with one 1,4-DITFB in a face-to-face style through π$\cdots$π stacking. Such molecular pairs are linked together by I$\cdots$I and C–I$\cdots$π halogen bonds, generating a particular zigzag chain with the iodine$\cdots$iodine chain in the middle and pyrene molecules on both sides (Fig. 26). In this way, 1,4-DITFB molecules function not only as an isolator to hinder the pyrene molecule from aggregating, but also as a heavy-atom disturber to generate phosphorescence emission from pyrene. The co-crystal obeys a bi-exponential model with an average lifetime of 0.574 ms. Furthermore, similar supramolecular chains were found in the phosphorescent co-crystals (Triphenylene)$\cdot$(1,4-DITFB) (*τ* = 9.7 ms) [137] and (Fluoranthene)$\cdot$(1,4-DITFB) (*τ* = 0.285 ms) [138]. Interestingly, (Triphenylene)$\cdot$(1,4-DITFB) undergoes a polymorphic phase transition caused by temperature.

Phosphorescent co-crystals can also be assembled by fluorene or its heterocyclic analogues (dibenzofuran and dibenzothiophene) with 1,4-DITFB based on C–I$\cdots$π interactions, providing 1D zigzag chains or grid-like chains [139]. The O or S atom in dibenzofuran or dibenzothiophene does not take part in any halogen-bonding interactions and the C–I$\cdots$π contacts are easier to form in the corresponding system, which has been confirmed by calculations of bonding energies. The phosphorescence spectra of the three co-crystals are greatly red-shifted by about 50–90 nm with regard to the free monomer in the *β*-cyclodextrin aqueous solution and all the decays obey a mono-exponential law with lifetimes of 0.34, 0.51 and 2.50 ms, respectively. In (Dibenzothiophene)$\cdot$(1,4-DITFB)_2_, the heavy-atom effect from the sulfur atom may be a contributing factor to the longer phosphorescence lifetime.

Later, the stoichiometric ratio of co-crystallization was utilized to tune the photophysical properties (fluorescence or/and phosphorescence) of the conjugated hydrocarbons diphenylacetylene as well as *trans*-stilbene by d’Agostino *et al.* [140]. The 1 : 1 molar ratio between the conjugated hydrocarbon and 1,4-DITFB facilitates the formation of a dual luminescent material with both fluorescence and phosphorescence emission, whereas the 1 : 2 stoichiometry makes their fluorescence suppressed so that only phosphorescence is observed. Comparatively speaking, the 1 : 2 co-crystals have lower emission quantum yields and shorter phosphorescence lifetimes, on account of the increased T_1_ → S_0_ radiative deactivation rate that is induced by the doubled heavy-atom/hydrocarbon stoichiometry.

### Organometallic complexes as the halogen-bonding acceptors

**Figure 27.**
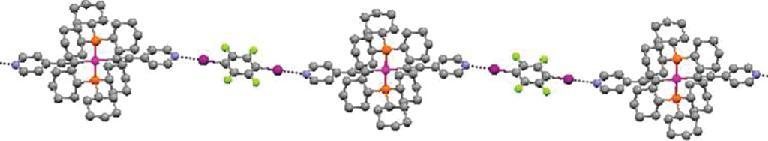
Halogen-bonding infinite chain of the co-crystal between *trans*-[Pt(PCy_3_)_2_(C≇C-4-Py)_2_] and 1,4-DITFB. Reprinted with permission from reference [141]. Copyright 2012 American Chemical Society.

Organometallic complexes are promising building blocks for the construction of multicomponent supramolecular assemblies. A novel co-crystal yielded upon isothermal evaporation of the CCl_3_ solution containing *trans*-[Pt(PCy_3_)_2_(C≇C-4-Py)_2_] (Cy = cyclohexyl) and 1,4-DITFB at room temperature, in which the Pt(II) ion has a rigid square-planar coordination environment [141]. The extremely strong C–I$\cdots$N interactions are primarily responsible for the self-assembly process (d_I$\cdots$N _= 2.698 Å, ∠C–I$\cdots$N = 177.6°), leading to the infinite supramolecular chains (Fig. 27). The broadband dielectric spectroscopic measurements, carried out at a temperature from 25 to 155°C and frequency from 10^−2^ to 10^7^ Hz, show that the co-crystal exhibits a real component of dielectric permittivity (*ϵ*^′^) that is remarkably lower than that of SiO_2_. Thereafter, a Pt(IV) complex with octahedral coordination geometry, PtMe_3_I(Cl-TPy) (Cl-TPy = 4^′^-chloro-2,2^′^:6^′^,2^″^-terpyridine), was exploited as the halogen-bonding acceptor, affording a discrete trimeric structure through the halogen-bonding interactions between uncoordinated pyridine nitrogen and iodine atoms from 1,4-DITFB [142]. The Pt-bound iodine is not involved in the halogen-bonding interaction, but results in a weak hydrogen bond with the solvent CCl_3_ instead.

**Figure 28.**
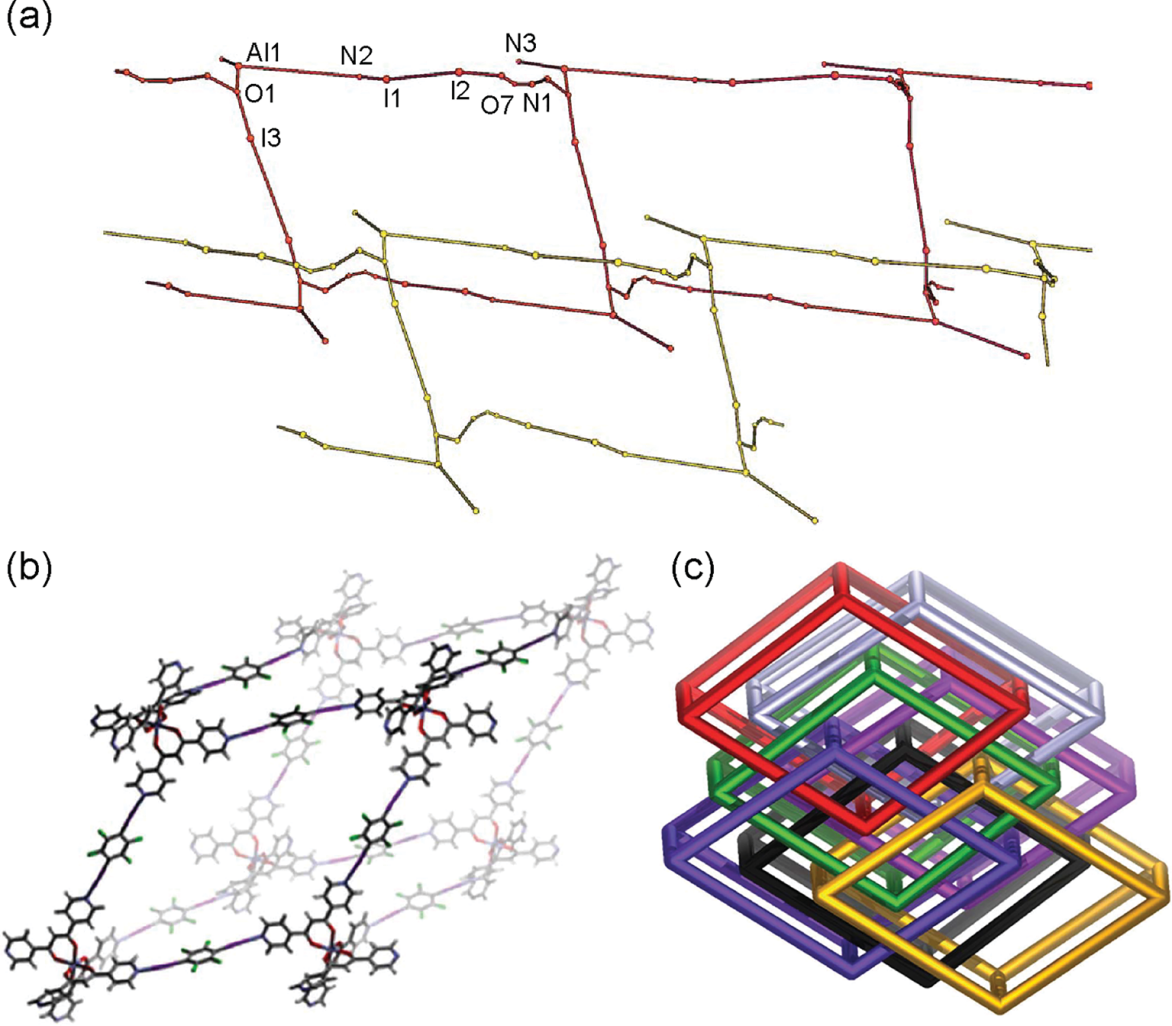
(a) Topology investigation of [Al(PyPDONE)_3_]_2_$\cdot$(1,4-DITFB)_3_$\cdot$2H_2_O with GTECS3D. (b) Repeating rhombohedral subunits in the 3D halogen-bonding network of [Fe(DPyPDONE)_3_]$\cdot$(1,4-DITFB)_3_$\cdot$0.5H_2_O. (c) Schematic representation of the 7-fold interpenetrated networks in [Fe(DPyPDONE)_3_]$\cdot$(1,4-DITFB)_3_$\cdot$0.5H_2_O. Reprinted with permission from references [48] and [143]. Copyright 2013 and 2018 Royal Society of Chemistry.

The octahedral complex Fe(PyPDONE)_3_ takes carbonyl oxygen as the coordination atom, leaving peripheral pyridine nitrogen as the potential halogen-bonding binding site [48]. Two of three pyridine nitrogen atoms are halogen bonded to I atoms of 1,4-DITFB to offer a 1D chain, with N$\cdots$I distances of a little less than 2.8 Å that indicate that metal coordination leads to shorter and stronger halogen-bonding interactions. However, isostructural Al(PyPDONE)_3_$\cdot$3H_2_O acts differently [48]: only one pyridine nitrogen atom is engaged in the halogen-bonding interaction with 1,4-DITFB, while a carbonyl oxygen atom along with solvent water also conducts as a halogen-bonding acceptor, codetermining the formation of 2D grid-like networks in combination with an O–H$\cdots$N hydrogen bond between the water molecule and another pyridine nitrogen (Fig. 28a). Lately, the first neutral metal-containing 3D halogen-bonding network with an *α*-Po **pcu** (primitive cubic unit) topology (4^12^$\cdot$6^3^) has been reported by Clegg and co-workers based on a six-coordinated Fe(DPyPDONE)_3_ (DPyPDONE = di(4-pyridyl)propane-1,3-dione) [143]. Each of the six pyridyl N atoms is linked to I atoms from six different 1,4-DITFB molecules, producing the [Fe(DPyPDONE)_3_]_8_(1,4-DITFB)_12_ subunit, which encapsulates a volume of ∼16 600 Å^3^ that is occupied by another six identical networks (Fig. 28b). In this way, a 7-fold interpenetrated network structure appears and the high degree of interpenetration probably results from the large aspect ratio of the 1,4-DITFB linkers (Fig. 28c).

The cobalt(II) complex, Co(DBM)_2_(Morpholine)_2_ (DBM = dibenzoylmethane), contains morpholine oxygen atoms as potential halogen-bonding acceptors and can form a co-crystal [Co(DBM)_2_(Morpholine)_2_(1,4-DITFB)_2_]$\cdot$(1,4-DITFB)_4_ with 1,4-DITFB [144]. Each Co(DBM)_2_(Morpholine)_2_ molecule is attached to two 1,4-DITFB molecules through a short O$\cdots$I separation of 2.81 Å, giving rise to the [Co(DBM)_2_(Morpholine)_2_(1,4-DITFB)_2_] substructure. The resulting assemblies stack in the [110] direction via C–I$\cdots$π contacts (3.49 Å) between the phenyl rings and 1,4-DITFB, providing a 1D grid-like halogen-bonding chain. Simultaneously, two other isostructural complexes [Co(DBM)_2_(Tmor)_2_(1,4-DITFB)_2_]$\cdot$(1,4-DITFB)_4_ and [Ni(DBM)_2_(Morpholine)_2_(1,4-DITFB)_2_]$\cdot$(1,4-DITFB)_4_ were achieved by a similar synthetic method [144].

With regard to the complexes M(acac)_2_ (M = Cu, Pd, Pt; Hacac = acetylacetone), oxygen atoms from the only ligand *β*-diketone were exploited to interact with I atoms of 1,4-DITFB, favoring the formation of a 1D linear chain through intermolecular bifurcated C–I$\cdots$*μ*_2_-(O,O) halogen bonds [145]. The I$\cdots$O distances are 3.354, 3.255 and 3.248 Å, respectively; the contact angles ∠C–I$\cdots$O are 157.07, 155.78 and 155.86°, correspondingly. The slightly larger I$\cdots$O separations in [Cu(acac)_2_]$\cdot$(1,4-DITFB) are probably ascribed to the Cu center with a higher charge density, which makes the nucleophilic property of adjacent oxygen atoms weaker.

In Cinčić’s research team, halogen-bonding metal-organic co-crystals were designed based on Schiff base complexes with pendant acetyl groups, M(NMAPE)_2_ (M = Cu, Ni; HNMAPE = (*E*)-1-(4-((2-hydroxynaphthalen-1-yl) methyleneamino)phenyl)ethanone), where unsaturated Cu(II) or Ni(II) ion adopts a square-planar geometry [146,147]. Each M(NMAPE)_2_ interacts with two 1,4-DITFB molecules via two acetyl oxygen atoms with O$\cdots$I distances of 3.084 and 3.117 Å, respectively, generating 1D zigzag halogen-bonding chains. Unlike the imine in the Schiff base complexes M(NMAPE)_2_ above, the nitrogen atoms in tetra-imino ferrocenophane can be reliably utilized as the halogen-bonding acceptors for the iodine atoms of 1,4-DITFB [148]. Specifically, only two of the four imine nitrogen atoms take part in the C–I$\cdots$N interactions that drive the co-crystallization process and promote the formation of polymeric chains. Mössbauer spectroscopy reveals the sole presence of low-spin Fe(II) and the temperature dependence of the magnetic susceptibility corresponds to a quasi-diamagnetic compound.

**Figure 29.**
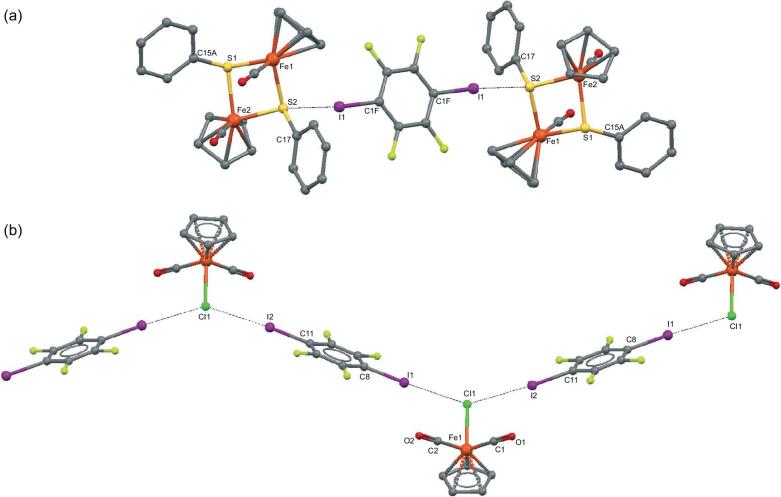
(a) Halogen-bonding trimolecular assemblies in {[CpFe(CO)(*μ*_2_-SPh)]_2_}_2_$\cdot$(1,4-DITFB). (b) Halogen-bonding polymeric chains in [CpFe(CO)_2_Cl]$\cdot$(1,4-DITFB). Reprinted with permission from reference [149]. Copyright 2018 Royal Society of Chemistry.

Recently, organometallic halides and chalcogenides, CpFe(CO)_2_X (X = Cl, Br, I, TePh, SPh; Cp = cyclopentadienyl), (TMCb)Co(CO)_2_I (TMCb = tetramethylcyclobutadiene) [149], CoL_2_Cl_2_ (L = 2,2^′^-BPy, 1,10-Phen) [150] and (BDBPMB)PdX (X = Cl, Br; BDBPMB = 2,6-bis[(di-*t*-butylphosphino)methyl] benzene) [151], were chosen to co-crystallize with 1,4-DITFB under slow vapor diffusion or liquid-assisted grinding conditions, yielding discrete assemblies through single C–I$\cdots$X halogen bonds or polymeric chains through bifurcated C–I$\cdots$X halogen bonds (Fig. 29). In the co-crystals of Fe and Co complexes, the 5–10 cm^−1^ hypsochromic shifts of the C = O stretching vibration bands can be explained by the obvious electron-withdrawing effect of the halogen bond, as supported by DFT calculations.

### Anions as the halogen-bonding acceptors

In general, anions do not merely balance the charge of cations, but also serve as halogen-bonding acceptors, whereas the cation takes virtually no active role in the formation of halogen bonds. As compared with neutral species, anions are better halogen-bonding acceptors since their Lewis basicity can be enhanced by the increased electron density on the electron-donor site.

#### Halide anion-templated (Cl^−^, Br^−^, I^−^, I_3_^−^) assembly of 1,4-DITFB

##### Onium ions (NR_4_^+^, PR_4_^+^) as the counterions.

**Figure 30.**
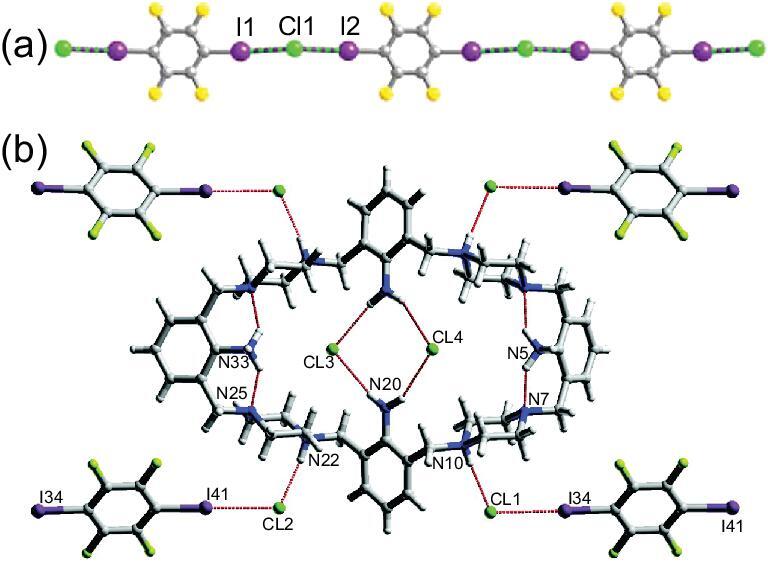
(a) Infinite halogen-bonding chain in [(Et_3_N-CH_2_Cl)Cl]$\cdot$(1,4-DITFB). (b) Halogen-bonding interactions in [(H_6_TAPC-2)Cl_6_]$\cdot$(1,4-DITFB)_2_. Reprinted with permission from references [153] and [107]. Copyright 2014 and 2010 American Chemical Society.

The reactions of ammonium or phosphonium chlorides (Me_4_NCl [152], (Et_3_N-CH_2_Cl)Cl [153], *n*-Bu_4_NCl [154], *n*-Bu_4_PCl [155], Ph_4_PCl [152]) and 1,4-DITFB afford several heteromeric three-component co-crystals. Each ditopic chloride ion links two neighboring 1,4-DITFB molecules through C–I$\cdots$Cl^−^ interactions to produce an infinite supramolecular chain (Fig. 30a), except one case of a discrete termolecular structure in (Ph_4_PCl)_2_$\cdot$(1,4-DITFB) that is ascribed to a monotopic Cl^−^ anion. Similar halogen-bonding chains appear in their bromide analogues ((Me_4_NBr)$\cdot$(1,4-DITFB) [152], [(Et_3_N-CH_2_Br)Br]$\cdot$(1,4-DITFB) [153], (*n*-Bu_4_NBr)$\cdot$(1,4-DITFB) [154,155] and (*n*-Bu_4_PBr)$\cdot$(1,4-DITFB) [155]) while trimeric assemblies occur in (EtPh_3_PBr)_2_$\cdot$(1,4-DITFB) [155] and (Ph_4_PBr)_2_$\cdot$(1,4-DITFB) [152]. Additionally, a 3D supramolecular network was observed in (Ph_4_PBr)_2_$\cdot$(1,4-DITFB)_3_ where tritopic bromide ions concatenate three 1,4-DITFB molecules through C–I$\cdots$Br^−^ halogen bonds [152]. The C–I$\cdots$Br^−^ interactions generally show longer distances (3.2–3.4 Å) than C–I$\cdots$Cl^−^ (3.05–3.25 Å) [153].

An extension of this work to cyclic cations was reported by Rissanen and co-workers, offering three novel co-crystals: [(H_3_TAPC-1)Cl_3_]$\cdot$(1,4-DITFB)_6_, [(H_6_TAPC-2)Cl_6_]$\cdot$(1,4-DITFB)_2_ [107] and [(H_4_ChAR)Br_4_]$\cdot$(1,4-DITFB)_4_ (H_4_ChAR^4+^ = protonated *N*-cyclohexyl ammonium resorcinarene) [156]. Herein, halide anions (Cl^−^ or Br^−^) are attached to large-molecular-weight polyammonium cations by N^+^−H$\cdots$X^−^ hydrogen bonds and halogen bonded to 1,4-DITFB via C–I$\cdots$X^−^ interactions, resulting in three-component C–I$\cdots$X^−^$\cdots$H–N^+^ synthons that drive the self-assembly processes (Fig. 30b). Halogen bond in conjunction with hydrogen bond provides an excellent route to elaborate and complex 3D supramolecular architectures.

When compared with chloride and bromide ions, iodide anions from Me_4_NI [152], *n*-Bu_4_NI [157] or Ph_4_PI [152] readily form trifurcated halogen bonds with the iodine atoms from 1,4-DITFB, thereby generating high-dimensional (2D or 3D) supramolecular networks or 1D ladder-like chains. A larger cation such as [(CH_3_)_3_N(CH_2_)_10_N(CH_3_)_3_]^2+^ changes the bonding mode above and only the single C–I$\cdots$I^−^ halogen bonds are found between I^−^ ions and 1,4-DITFB, leading to low-dimensional discrete termolecular assemblies [158]. If containing halogen atoms (e.g. [I(INPPh_3_)_3_]^+^), the cation not only balances the charge, but also together with 1,4-DITFB functions as the halogen-bonding donor of the tridentate I^−^ anion, yielding a zigzag chain [159].

**Figure 31.**
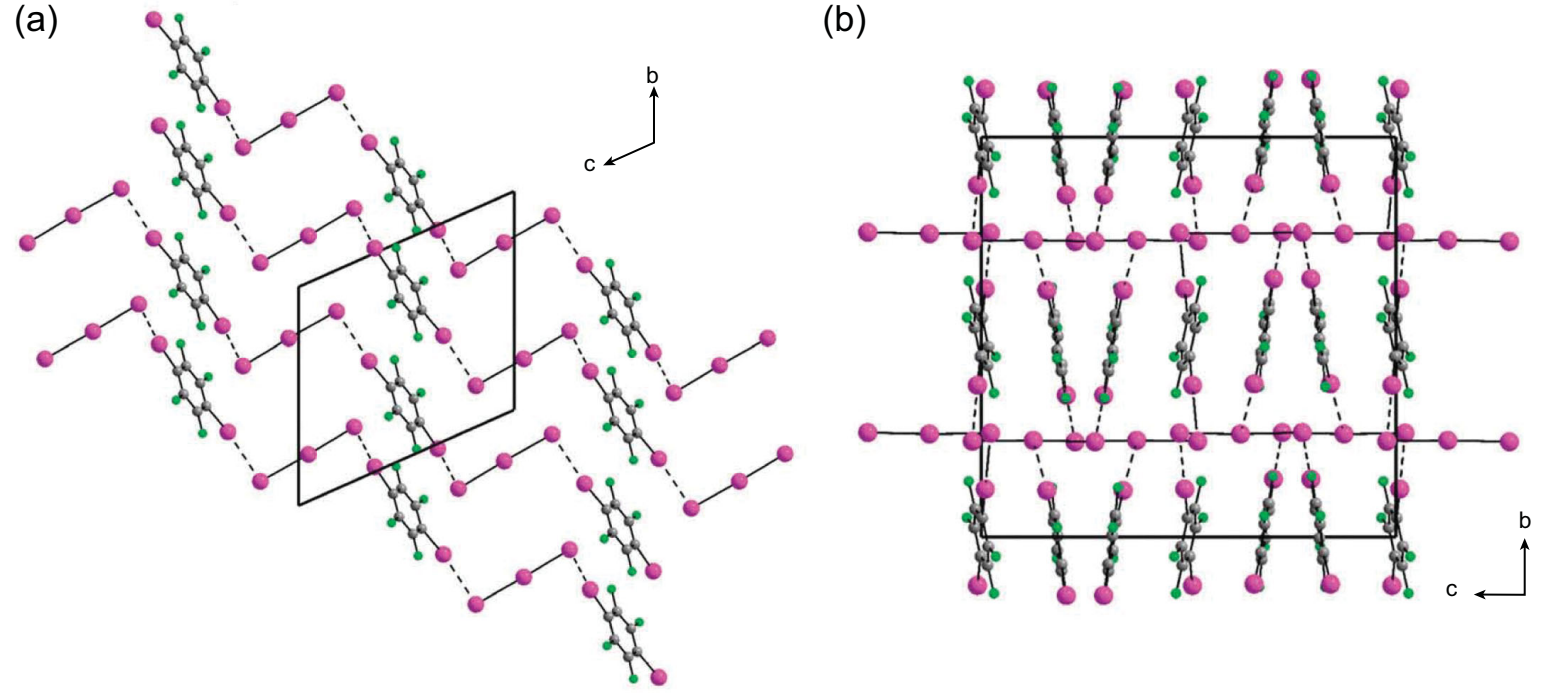
(a) Halogen-bonding zigzag chains in (MePh_3_PI_3_)$\cdot$(1,4-DITFB). (b) 3D supramolecular networks in (MePh_3_PI_3_)_2_$\cdot$(1,4-DITFB)_3_. Reprinted with permission from reference [160]. Copyright 2018 Royal Society of Chemistry.

The linear triiodide anion (I_3_^−^) with directionality has the capability to participate in the formation of halogen bonds, though the negative charge is more diffuse than that of a monoatomic iodide anion. In the 1 : 1 co-crystal (MePh_3_PI_3_)$\cdot$(1,4-DITFB) [160], both the terminal iodine atoms of I_3_^−^ are involved in the C–I$\cdots$I^−^ halogen bonds (d_I$\cdots$I^−^_ = 3.489 and 3.648 Å, ∠C–I$\cdots$I^−^ = 171.9° and 163.9°, respectively), forming a zigzag chain (Fig. 31a). With respect to the 2 : 3 co-crystal (MePh_3_PI_3_)_2_$\cdot$(1,4-DITFB)_3_, the central iodine atoms besides the terminal ones are halogen bonded to 1,4-DITFB, extending the zigzag chain into a 3D supramolecular network (Fig. 31b).

##### Metal complex cations as the counterions.

**Figure 32.**
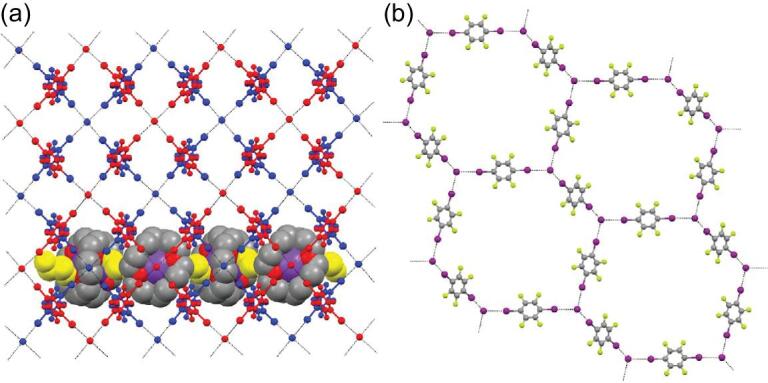
(a) Two adjacent layers of 2D (4,4) grid-like sheets in (K.2.2.2.⊂KBr)$\cdot$(1,4-DITFB)_2_$\cdot$EtOH. (b) 2D (6,3) honeycomb-like anionic supramolecular network of (K.2.2.2.⊂KI)_2_$\cdot$(1,4-DITFB)_3_. Reprinted with permission from reference [157]. Copyright 2010 Elsevier.

The cryptate K.2.2.2.⊂KX (X = Br^−^ or I^−^) can also work as a source of naked halide anions, in which K.2.2.2. is 4,7,13,16,21,24-hexaoxa-1,10-diazabicyclo[8,8,8]hexacosane (HDABCH). Based on cryptates, three heteromeric three-component systems were achieved by Resnati's group, namely (K.2.2.2.⊂KBr)$\cdot$(1,4-DITFB)_2_$\cdot$EtOH, (K.2.2.2.⊂KI)_2_$\cdot$(1,4-DITFB)_3_ and (K.2.2.2.⊂KI)_2_$\cdot$(1,4-DITFB)_3_$\cdot$2CCl_4_ [157]. In the bromide, tetradentate Br^−^ ions bridge four different 1,4-DITFB molecules via C–I$\cdots$Br^−^ halogen bonds with distances of 3.280 and 3.350 Å, and sit at the nodes of a somewhat distorted square, giving slightly undulated 2D (4,4) grid-like sheets in which the I$\cdots$Br^−^$\cdots$I angles are 85.53 and 90.97°, respectively (Fig. 32a). The cations locate perfectly at the center of the square and consequently interpenetration is prevented.

As for (K.2.2.2.⊂KI)_2_$\cdot$(1,4-DITFB)_3_, tridentate iodide anions behave as the *μ*_3_-bridges and connect three different 1,4-DITFB molecules into regular 2D (6,3) honeycomb-like sheets via C–I$\cdots$I^−^ halogen bonds (Fig. 32b), wherein the I$\cdots$I^−^$\cdots$I angles are close to the tetrahedral geometry (119.45°, 107.62 and 101.65) and the I$\cdots$I^−^ contacts span the range of 3.352–3.379 Å. Two such sheets interpenetrate each other with a dihedral angle of 62.7°, creating a 3D supramolecular network. In its solvate (K.2.2.2.⊂KI)_2_$\cdot$(1,4-DITFB)_3_$\cdot$2CCl_4_, each I^−^ ion interacts with three iodine atoms from different 1,4-DITFB molecules, inducing the formation of highly undulated 2D (6,3) honeycomb-like sheets that are attributed to the distorted I$\cdots$I^−^$\cdots$I angles (72.33, 112.60 and 123.68°). Two hexagonal networks are linked together by CCl_4_ solvent molecules through I^−^$\cdots$Cl–CCl_2_–Cl$\cdots$I^−^ halogen-bonding interactions, resulting in a 3D diamond network (6^6^-**dia**) with 2-fold interpenetration. In the overall structure, the iodide anion is in a pentadentate mode and its electron density is distributed over five halogen bonds, which are responsible for the longer I^−^$\cdots$X (X = Cl or I) contacts ranging from 3.435 to 3.587 Å.

#### Thiocyanate and selenocyanate anion-templated (SCN^−^, SeCN^−^) assembly of 1,4-DITFB

Co-crystallization of ammonium thiocyanates (Me_4_NSCN [161], Et_4_NSCN or *n*-Bu_4_NSCN [162]) with 1,4-DITFB allowed the isolation of three different supramolecular salts. X-ray crystallographic analyses combined with theoretical calculations reveal the bidentate characteristic of the SCN^−^ anion where the ends of both S and N are engaged in halogen-bonding interactions, giving rise to 1D zigzag chains or high-dimensional (2D or 3D) supramolecular networks. Short I$\cdots$N/S contacts are observed with the C–I$\cdots$N/S angles near linear, but the I$\cdots$N≇C angles deviate from linearity and the I$\cdots$S–C angles are even close to 90°. Furthermore, ammonium selenocyanate (Me_4_NSeCN) was utilized to prepare a new supramolecular complex (Me_4_NSeCN)(1,4-DITFB)_2_, in which SeCN^−^ anion binds in a similar fashion to SCN^−^ described above [161]. Multinuclear solid-state magnetic resonance spectroscopy of these complexes shows that ^13^C chemical shifts of the thiocyanates slightly increase and yet ^15^N chemical shifts decrease in contrast with reference compounds with simple counterions, indicating the existence of halogen bonds. The opposite trends are captured for the selenocyanates and more substantial changes are found in the pseudo unique principal component of the ^77^Se chemical shift tensor as well as the ^77^Se isotropic chemical shift.

#### Tetrahedral oxyanion-templated (ClO_4_^−^, IO_4_^−^, ReO_4_^−^) assembly of 1,4-DITFB

The potential of tetrahedral oxyanions as halogen-bonding acceptors was investigated by Resnati *et al.* Self-assembly of *n*-Bu_4_NXO_4_ (XO_4_^−^ = ClO_4_^−^, IO_4_^−^, ReO_4_^−^) with 1,4-DITFB affords the undulated (6,3) networks via C–I$\cdots$O halogen bonds wherein tridentate anions sit at the network nodes bridged by bidentate 1,4-DITFB molecules and the ammonium cations occupy the space that is encircled by the (6,3) frames [163]. The I$\cdots$O distances span from 2.864 to 3.264 Å and the C–I$\cdots$O angles are in the range of 160.53–172.35°. The remarkably similar structures among the three oxyanions prompted them to synthesize several mixed crystals (e.g. (*n*-Bu_4_N^+^)$\cdot$(ClO_4_^−^)_0.72_$\cdot$(ReO_4_^−^)_0.28_$\cdot$(1,4-DITFB)_1.5_), which are isostructural with the halogen-bonding supramolecular salts discussed above. Thereinto, two different oxyanions statistically occupy the nodes of (6,3) networks with ratios, even though they have quite different sizes. Tetrahedral oxyanions hence proved to be effective and general tectons in the anion-templated assembly, which is driven by halogen-bonding interactions.

## CONCLUSION

In this review, an attempt has been made to sketch out the efforts to obtain multicomponent supramolecular complexes through the co-crystallization of 1,4-DITFB with a variety of halogen-bonding acceptors in the range from neutral Lewis bases (nitrogen-containing compounds, N-oxides, chalcogenides, aromatic hydrocarbons and organometallic complexes) to anions (halide ions, thio/selenocyanate ions and tetrahedral oxyanions). The examples reviewed here illustrate that halogen bonds have a vital role in co-crystallizing processes, exhibiting a wide diversity of impressive supramolecular architectures (for instance, dimers, trimers, tetramers, pentamers, heptamers, 13-molecule finite chains, 1D infinite chains, highly undulated infinite ribbons, 2D and 3D networks).

It is not easy to chase down common features among this large family of halogen-bonding supramolecular complexes based on 1,4-DITFB and yet their topological structures can be modulated by the different halogen-bonding acceptors. In a general way, anionic acceptors form furcated halogen bonds with iodine atoms from 1,4-DITFB, readily giving rise to high-dimensional (2D or 3D) supramolecular networks. Furthermore, neutral species with multiple potential binding sites, for example tetrapyridyl compounds, favor the formation of high-dimensional supramolecular structures as well.

Many interesting physicochemical properties are found in these co-crystals, such as fluorescence, phosphorescence, magnetism, dielectric and non-linear optical properties, as well as liquid crystals and supramolecular gels. Among them, π-conjugated aromatic hydrocarbons can be exploited to construct phosphorescent materials, while the use of N-oxides has made the achievement of multicomponent magnetic complexes possible. Moreover, co-crystals of pyridine derivatives with alkoxy chains exhibit liquid crystalline behavior.

In addition, some co-crystals based on pyridine derivatives are able to be applied in different photoelectric devices, e.g. optical waveguide, laser, optical logic gate and memory. The microplate (MPyIMP)$\cdot$(1,4-DITFB) displays a self-waveguided edge emission, strong 1D field confinement and FP-type resonance, realizing organic co-crystal lasers. Besides, the asymmetric optical waveguide makes the co-crystal of OPV-3 suitable for the construction of a microscale optical logic gate, contributing to the development of organic integrated photonics. Two other supramolecular assemblies from isomeric PVA enrich the library of piezochromic materials for haptic memory.

Although the utilization of 1,4-DITFB in the co-crystallization area has been ongoing for almost two decades, the relationship among molecular structures, assembly processes and functional properties has not been yet well understood. Exploration of photo-electro-magnetic functional materials has only just begun and even less is known about photoelectric devices as well as soft matter. From the point of view of crystal engineering and supramolecular chemistry, these co-crystals will offer helpful information for further design and investigation of the elusive family of halogen-bonding complexes. We believe that multicomponent supramolecular complexes assisted by halogen bonds should have a great potential and bright future in functional materials and device applications.
